# Supplementary material for: Estimating smoking prevalence in general practice using data from the Quality and Outcomes Framework (QOF)
Source: BMJ Open. 2014 Jul 16;4(7):e005217. doi: 10.1136/bmjopen-2014-005217 (PMC4120299; doi:10.1136/bmjopen-2014-005217)
Supplement: Author's manuscript [file bmjopen-2014-005217.draft_revisions.pdf]

Estimating smoking prevalence in general practices: an evaluation of QOF (Quality and Outcomes Framework) data

Journal:	*BMJ Open*
Manuscript ID:	bmjopen-2014-005217
Article Type:	Research
Date Submitted by the Author:	07-Mar-2014

Complete List of Authors:	Honeyford, Kate; University of Leicester, Health Sciences<br>Baker, Richard; University of Leicester, Health Sciences<br>Bankart, M. John; Keele University, Institute of Primary Care and Healthe Sciences<br>Jones, David; University of Leicester, Health Sciences

**Primary Subject Heading**:	Epidemiology
Secondary Subject Heading:	Health services research, Public health, Smoking and tobacco
Keywords:	EPIDEMIOLOGY, STATISTICS & RESEARCH METHODS, PUBLIC HEALTH, PRIMARY CARE

SCHOLARONE™ Manuscripts

TITLE

Estimating smoking prevalence in general practices: an evaluation of QOF (Quality and Outcomes Framework) data

AUTHORS' INFORMATION

Corresponding author:

Kate Honeyford MSc
Adrian Building,
University Road,
Leicester
LE1 7RH
ceh28@le.ac.uk
Tel: 0116 229 7254/7255
Fax: 0116 229 7250

Co-authors:

Professor Richard Baker, Dept of Health Sciences, University of Leicester, Leicester, UK.
Dr M. John G. Bankart, Institute of Primary Care and Health Sciences, Keele University, Keele, UK.
Professor David R Jones, Dept Health Sciences, University of Leicester, Leicester, UK.

KEYWORDS

Smoking/epidemiology
Population surveillance
Primary Health Care
Cardiovascular disease

WORD COUNT

2987 words

For peer review only

ABSTRACT

Background

Reliable estimates of smoking prevalence in general practice populations are useful when comparing practice-level health outcomes, and informing intervention targeting in primary care.

This paper explores whether data based on patients' medical records, published as part of the Quality and Outcomes Framework (QOF), can be used to estimate smoking prevalence within practice populations, and evaluates the usefulness of these estimates.

Methods

Cross-sectional analysis of 215 practices in three East Midlands PCTs. Simple manipulations of QOF indicator data provide smoking prevalence estimates in general practice populations and among patients with chronic conditions. Bland-Altman limits of agreement between estimates from the integrated household survey (IHS) and aggregated QOF-based estimates were calculated. The impact of including smoking estimates in negative binomial regression models of counts of premature CHD deaths was assessed.

Results

Median smoking prevalence in the practice populations for 2012/13 was 19.2% (range 5.8% to 43.0%). There was good agreement (mean difference: 0.39%; 95% limits of agreement (-3.77, 4.55)) between IHS estimates for local authority districts and aggregated QOF register estimates. Smoking prevalence estimates in those with chronic conditions are lower than for the general population (mean difference -3.05%), but strongly correlated (R_p=0.74, p<0.0001). An important positive association between premature CHD mortality and smoking prevalence was shown when smoking prevalence was added to other population and service characteristics.

Conclusions

Published QOF data allow useful estimation of smoking prevalence within practice populations and in those with chronic conditions; the latter estimates may sometimes be useful in place of the former.

Strengths and Weaknesses

- This paper clearly demonstrates that useful estimations of smoking prevalence within practice populations can be calculated from routine data published through the Quality and Outcomes Framework (QOF).
- Our analysis shows that estimates of smoking prevalence in those with chronic conditions can be used in some situations in place of an estimate for the general population, if this is not available.
- QOF data rely on self-reported smoking status, recorded in the previous 27 months, which may underestimate smoking status or the effectiveness of interventions.
- This study does not have access to individual patient data limiting our understanding of patients who do not have smoking status recorded and the possible impact of missing data on estimates of smoking prevalence.

For peer review only

For peer review only

Background

Despite smoking prevalence in England falling "below 20% for the first time in 80 years",[1] reducing smoking remains a key public health priority in England as in many countries, with local authorities and primary care services being expected to play a key role in local tobacco control services.[2] In addition, clinical commissioning groups (CCGs), membership organisations responsible for planning, organising and purchasing nationally funded healthcare within their local areas have their own health targets. Reducing smoking prevalence is a key component of many targets, for example reducing chronic obstructive pulmonary disease (COPD) outcomes [3] and reducing inequalities in coronary heart disease (CHD).[4] Access to reliable estimates of smoking prevalence in practice populations is useful to assess need, inform targeting of interventions delivered through primary care and to evaluate those interventions. In addition, research into a variety of health outcomes and their associations with primary care needs to take characteristics of the practice populations into account. Currently a variety of measures of smoking prevalence in practice populations are being used [5-6] and some do not include smoking,[7-9] despite the recognized associations between smoking prevalence and a range of chronic conditions.[10]

National Survey

In England, there are various national surveys of smoking prevalence including the Health Survey for England;[11] the General Lifestyle Survey;[12] the Smoking Toolkit Study (STS) and the Integrated Household Survey (IHS). The IHS began in 2009; a composite survey including questions on smoking habits (over 420,000 adults in 2011). IHS statistics are designated as experimental, in a 'testing phase' and not yet fully developed,[13] but estimates are available for local authorities. None of these surveys aims to establish the smoking prevalence within practice populations.

Patient records

Analyses of individual patient records, using the THIN (The Health Improvement Network) [14] and QRESEARCH [15] databases, provide strong evidence that smoking status within primary care medical records could be used to monitor national smoking patterns. There was good agreement between smoking prevalence based on medical records in the THIN database and those predicted by GHS; 22.4% compared to 21.8% respectively in males; 18.9% compared to 20.2% respectively in females.[16] Estimates of smoking prevalence based on the medical records in the QRESEARCH database has also shown good agreement with national surveys, in this case the Health Survey for England.[17]

QOF

The national Quality and Outcomes Framework (QOF) was introduced in England in 2004 to improve the quality of primary care for patients. Since its inception QOF has included indicators relating to smoking.[8] The underlying aim of these indicators has not changed over the years; a) practices should record smoking status in patient notes and, b) for those who smoke, smoking cessation advice/support/treatment should have been offered. Until 2012/13 the focus was on targeting smoking cessation advice to those with chronic conditions. Table 1 summarises QOF smoking indicators 2006/7 – 2014/15.

The QOF indicators have not been designed to determine smoking prevalence within the practice population; indeed it is clearly stated that 'QOF provides no information on numbers of smokers and non-smokers',[19] attributing this mainly to the condition-specific nature of the indicator. The wording has not changed since the inclusion of the two new indicators which apply to the general population and are not condition-specific.

Objective

In this paper we aim to explore to what extent underlying data published as part of QOF can be used to estimate smoking prevalence within practice populations and to evaluate the usefulness of such estimates.

For peer review only

METHOD

Sample

All practices within three primary care trusts (PCTs) (2006/07 to 2011/12) in the East Midlands were eligible for inclusion in the study. 215 practices with QOF data available for the seven financial years were included in the analysis. 14 practices were excluded because they lacked data for all seven years. One practice was excluded from the study as it served a restricted practice list; another practice was excluded from the study as 2012/13 QOF data strongly suggested an error.

Manipulation of QOF data

In order to estimate smoking prevalence, both the number of people who smoke and the population from which this number is drawn must be known. Two key QOF indicators are used in the calculations of smoking prevalence in the total practice population:

- SM07 'The percentage of patients aged 15 years and over whose notes record smoking status in the preceding 27 months'.
- SM08 'The percentage of patients aged 15 years and over who are recorded as current smokers who have a record of an offer of support and treatment within the preceding 27 months'.

In this analysis the denominator of the smoking cessation indicator was used as a measure of the number of people who smoke; the population was based on the denominator of the smoking status indicator.
In addition indicators of a similar nature were included but applying to those with any, or any combination, of a range of QOF specified chronic conditions (SM05 and SM06).

QOF data can be downloaded from the Health and Social Care Information Centre website containing information for all practices in a region;[20] Table 2 illustrates the type of data available.
Using the data given for these indicators it is possible to estimate the smoking prevalence in a practice population. For example, for practice A the denominator for SM07 is 3721 – the number of people for whom smoking status should be determined. This includes the whole practice population aged over 15, with the exception of people who have joined the practice in the three months prior to the data extraction point and patients who refuse to provide their smoking status. The denominator for SM08 is 1129 - indicating that there are 1129 registered patients recorded as smokers. Hence smoking prevalence can be estimated as 1129/3721 or 30.3%, see Table 2. This method was used to estimate smoking prevalence for the total practice population in 2013/14 and, using appropriate indicators, for those with chronic conditions from 2006/07 to 2013/14.

In addition, the percentage of the practice population with a chronic condition was determined using the denominator of SM07 as a measure of the practice population and SM05 as a measure of the practice population with a chronic condition.

IHS data

IHS smoking prevalence data are published for the financial years 2009/10 and 2011/12 at various geographical levels including counties, local authority districts and unitary authorities.[21] Data at practice level have been combined to create district level QOF-based estimates based on the post code of the practice to allow comparisons with IHS estimates.

Modelling

To determine the importance of being able to determine an estimate of smoking prevalence for practice populations, the estimate of smoking prevalence was included in a model to determine the associations of premature CHD (under 75) mortality with various population and service characteristics; the methods are described by Honeyford et al.[**Error! Reference source not found.**] Here, counts of premature CHD deaths (between April 2006 and March 2009) were modelled using negative binomial regression, using the same explanatory variables but including estimated smoking prevalence for those with chronic conditions based on QOF 2006/07. Service and population characteristics derived from QOF registers from 2006/07 were originally selected for inclusion in the study but an estimate of smoking prevalence for the general population was not available for this year.

For peer review only

RESULTS

Estimation of overall smoking prevalence using QOF smoking indicators 2012/13

The median underlying achievement for the recording of smoking status in the total practice population was 88.1% in 2012/13 (IQR: (83.7, 91.0)). The median estimate of smoking prevalence in practice populations was 19.2%, ranging from 5.8% to 43.0% (IQR: (15.1%, 22.9%)). Estimates of smoking prevalence were in line with estimates derived from the IHS. Aggregating over the total area, smoking prevalence was 19.5%, compared to 19.3% when IHS district level data were aggregated over the same area. When practice data were combined to give estimates of smoking for local authority districts there was a strong positive correlation (R_p=0.86, p<0.0001) and good agreement (mean difference: 0.39%; 95% limits of agreement (-3.77, 4.55)) between estimates based on QOF registers and IHS estimates (Fig. 1).[22]

Estimates of smoking prevalence in those with chronic conditions using QOF smoking indicators 2012/13

The underlying achievement for recording smoking status in those with chronic conditions was higher than for the total practices population (96.6% IQR (95.0, 97.7)). The median practice based estimate for those with any or any combination of a specific list of chronic conditions was 15.4% (IQR: 12.6% to 19.4%), ranging from 7.1% to 51.5%. When the estimates of prevalence for those with chronic conditions were aggregated into local authority districts, estimates were lower than IHS estimates for the majority of areas.

Association between smoking prevalence in the general practice population and those with chronic conditions.

Smoking prevalence in those with chronic conditions is lower than in the general practice population. The mean difference between the two estimates was -3.05% (95% limits of agreement: (-8.65, 1.56)).The Bland-Altman plot does not suggest a strong pattern, despite some evidence that the difference increases as the average increases (Fig. 2). There was a strong positive correlation (R_p=0.92, p<0.0001) between the overall estimate of smoking prevalence within a practice population and in those with chronic conditions. A regression model was developed to predict smoking prevalence in the general population based on the prevalence in those with chronic conditions; removal of outliers improved model fit.

Associations between recording of smoking status and prevalence

There was a strong positive correlation between recording of smoking status in the general population and in those with chronic conditions (underlying achievement for SM07 and SM05 respectively) (R_p=0.74, p<0.0001). There was no evidence of an association between smoking prevalence in the general population and recording of smoking status (R_p=-0.07, p=0.28) or the percentage with a chronic condition (R_p=0.03, p=0.67).

QOF smoking indicators 2006/07-2012/13

The estimates of smoking prevalence in those with chronic conditions have been consistent since 2006/07, with the median varying slightly during that time. Concordance was high between estimates for all years; Lin's concordance coefficient [23] was greater than 0.92 and mean difference was less than one in all cases (Table S1 in Appendix for more details).

Including smoking prevalence estimates in models of mortality

Table 3 shows incident rate ratios, 95% CIs and associated p values for the original and modified models. Inclusion of the smoking prevalence variable in the model reduced the strength of the associations between deprivation and premature mortality, and percentage white and premature mortality. A one unit increase in smoking prevalence was associated with an increase of 3.2% in expected premature CHD mortality count. If a practice with a moderately high smoking prevalence (75th percentile: 18.86%) is compared to one with a median level of smoking prevalence (15.09%), a difference of 11.69% in premature CHD mortality count can be expected, after adjusting for the other variables in the model.

Sensitivity analysis considering the impact of exception reporting indicates no impact on interpretation (see Doran for details of exception reporting [24]).

For peer review only

Discussion

Principal findings

These results show how QOF registers can be used to estimate smoking prevalence in practice populations and that these estimates are useful when analysing patterns of mortality.

When smoking prevalence is estimated in the general population using QOF indicators there is good agreement with estimates of IHS smoking prevalence for similar geographical areas.
QOF data can also be used to estimate smoking prevalence in those with chronic conditions, which is generally lower than smoking prevalence in the general population. There is good agreement between the estimates in successive years. The correlation between estimates of smoking prevalence in the general population in 2012/13 and those with chronic conditions is strong. These strong correlations suggest that the estimates based on previous years can be used in place of smoking prevalence in the general population for some purposes. Regression analysis suggests that smoking prevalence in those with chronic conditions can be used to predict smoking prevalence in the general practice population, for practices with a typical patient list.

When an estimate of smoking prevalence in those with chronic conditions was used in a study of the association between premature CHD mortality and various population and service characteristics an important positive association between CHD mortality and smoking prevalence was shown.

Strengths and weaknesses

The agreement between IHS based area estimates of smoking prevalence and estimates based on combining QOF data provides evidence to suggest that manipulating QOF data results is a useful measure of smoking prevalence within practice populations when compared to other available measures. This is supported by the work of Szatkowski et al [16] which found good agreement between national smoking prevalence predicted by patient records and the General Household Survey.
When comparing practices and analysing patterns across practices, it is important that the estimate is consistent across practices. The percentage of patients who do not have their smoking status recorded varies from 40% to less than 1%, the characteristics of these patients are not known. Recording of smoking status has been shown to vary between groups [17, 18, 25]; women, older people and those with chronic conditions were more likely to have their smoking status recorded. National surveys suggest that smoking rates are lower in these groups and therefore smoking prevalence from QOF may underestimate actual smoking prevalence. The implications of this will vary between practices, dependent on the proportion of these groups within their practice populations. Our analysis did not find an association between the percentage with a chronic condition and the recording of smoking status in the total population or the estimate of smoking prevalence.
QOF data are based on self-reported smoking status, which has been shown to be reliable in the general population,[25] but to underestimate smoking prevalence in pregnant women.[26] In addition, practices are only asked to record smoking status in the preceding 27 months, meaning the estimates may be useful in assessing need and analysing associations, but will have disadvantages in assessing the effectiveness of interventions, unless practices commit to more regular recording.
Practice level data have been aggregated to local authority districts based on practice postcode rather than patient postcodes; it is relatively common for practice postcodes to be used as a proxy for patient postcodes but when used to estimate deprivation has been found to underestimate relationships between deprivation and health outcomes.[27-28] Further work using individual patient records is necessary to analyse the frequency of recording of smoking status and the characteristics of patients for whom no smoking status is recorded or have been excluded on the basis of exception reporting.

Implications

Having estimates of the smoking prevalence in practice populations is important to those tasked with reducing smoking rates and improving the nation's health. CCGs and public health departments in local authorities need them to target smoking cessation and other additional resources. Understanding more about the patient populations would enable similar practices to be compared when considering differences in health outcomes and the apparent effectiveness of interventions.[26]

For peer review only - http://bmjopen.bmj.com/site/about/guidelines.xhtml

When estimates of smoking prevalence are included in the analysis of the associations between premature CHD mortality and practice population and service characteristics, there are reductions in magnitude of the incident rate ratios (IRRs) for both deprivation and percentage white. This suggests that these may be acting as surrogate markers of other lifestyle factors, such as smoking prevalence. Hence, the lack of reliable smoking information may be leading to relative over emphasis being placed on socio-economic deprivation, often described using an index of multiple factors. Similarly, it was found that social class was not linked to hospital admissions for stroke and CHD when rates were adjusted for various factors including smoking.[27] However, even with smoking prevalence included in the models, Brettell et al [29] found that increased deprivation was associated with higher heart failure admission rates, and Purdy et al [6] found that higher deprivation was associated with increased emergency admissions for myocardial infarction and angina. Unless we have reliable measures of smoking prevalence it is difficult to determine the relative importance of deprivation and other characteristics in explaining inequalities in a variety of health outcomes.

Smoking prevalence in those with chronic conditions is typically lower than in the general population. This may be due to diagnosis increasing motivation to quit smoking,[29] the increase in smoking cessation advice and support [30] or the age and gender profile of those with chronic conditions. Smoking prevalence in those with chronic conditions has not reduced over the seven year period, possibly suggesting that smoking cessation advice has limited effect, but may be due to the turnover of patients with chronic conditions as a result of both premature mortality and new diagnoses. A wide range of smoking cessation advice and support has recently been reviewed by Zwar et al;[31] consideration of how these impact on those with chronic conditions is recommended as a result of this finding.

QOF smoking indicators have changed since 2004 and continue to change. The introduction, in 2012/13, of an indicator which allows estimates of the smoking prevalence within the general population is useful for researchers as well as CCGs and public health officials. The removal of the indicator that covers the recording of smoking status in the total population from QOF in 2014/15 will impact on the methodology described in this paper, although the number of patients who are recorded as current smokers will continue to be available. The population of the practice will need to be used as the denominator in the calculation of smoking prevalence. It will be important to determine if the smoking status declines after the removal of the indicator; a recent study suggests that removal of indicators does not lead to a decline in clinical activities.[32]

Conclusion

Data published through QOF allow useful estimations of smoking prevalence within practice populations and in those with chronic conditions to be made. These estimates are important in developing our understanding of differences in health outcomes between practices, and are useful to both individual practices and CCGs when comparing practice level health outcomes, to assess need and to inform targeting. Revisions to QOF means that researchers will need to update methodology as indicators change.

For peer review only

Table 1 Summary of smoking indicators for which underlying achievement is published

General form of the indicator	Patient group	
	Patients with any, or any combination of the following conditions: coronary heart disease, stroke or TIA, hypertension, diabetes, COPD or asthma[1].	All patients aged 15 years+
**% of patients whose notes record smoking status[2]**	SM01: 2006/07 & 2007/08<br>SM03: 2008/09 – 2011/12<br>SM05: 2012/13<br>SMOK002: 2013/14 – 2014/15	Records 22: 2006/07 & 2007/08<br>Records 23: 2008/09 – 2011/12<br>SM07: 2012/13<br>SMOK001: 2013/14 – retired in 2014/15
**% of patients who are recorded as current smokers whose notes contain a record that smoking cessation advice or referral to a specialist service, where available, has been offered within the previous 15 months[3]**	SM02: 2006/07 & 2007/08<br>SM04: 2008/09 – 2011/12<br>SM06: 2012/13<br>SMOK005: 2013/14 – 2014/15	SM08: 2012/13<br>SMOK004: 2013/14 – 2014/15
**The practice supports smokers in stopping smoking by a strategy which includes providing literature and offering appropriate therapy.**		Information 5: 2006/07-2011/12<br>SMOK003: 2012/13 – 2014/15

[1]In 2008/09 CKD, asthma, schizophrenia, bipolar affective disorder or other psychoses were added to the list of chronic conditions and in 2012/13 PAD was added.

[2]For those with chronic conditions, the record must have been made in the past 15 months, reduced to 12 months in 2013/14, for all patients the period is 27 months, reduced to 24 months in 2013/14.

[3]In 2012/13 this changed to 'who have a record of an offer of support and treatment within the preceding 15 months', the period is 27 months for all patients, reduced to 12 months and 24 months respectively in 2013/14.

For peer review only

Table 2 Example of QOF data from 2012/13, showing how it can be used to calculate smoking prevalence for individual practices.

		Example practices				
QOF description	Interpretation for purposes of calculating smoking prevalence	A	B	C	D	E
SM07 Points		11	10.5	10.8	9.6	11
SM07 Numerator	Patients[1] whose notes contain a record of smoking status	3450	1319	6276	31948	6504
SM07 Denominator	Patients who are eligible to be included in this indicator[2]	3721	1497	7033	37654	7212
SM07 UA		92.70%	88.10%	89.20%	84.80%	90.20%
SM08 Points		12	9.9	12	8.9	12
SM08 Numerator	Patients who are recorded as current smokers and have a record of an offer of support etc	1024	325	1578	8439	2165
SM08 Denominator	Patients who are recorded as current smokers	1129	401	1586	10931	2373
SM08 UA		90.70%	81.00%	99.50%	77.20%	91.20%
	Calculation to determine percentage who are smokers SM08 den/ SM07 den	1129/3721	401/1497	1586/7033	10931/37654	2373/7212
	Estimate of smoking prevalence	30.30%	26.80%	22.60%	29.00%	32.90%

1[1]*Patients aged over 15*
[2]*For example patients who are newly registered with the practices (less than three months) are excluded from the indicator*

For peer review only

Table 3 Estimated incident rate ratios (IRRs) for premature (U75) CHD mortality count (n=215)[1].

	***without** smoking prevalence variable*				***with** smoking prevalence variable*		
**Explanatory variable**	***IRR***	***95% CI***	***p value***		***IRR***	***95% CI***	***p value***
Percentage white patients	1.007	(1.003, 1.012)	0.002		1.001	(0.995, 1.007)	0.657
Deprivation score (IMD 2007)	1.017	(1.011, 1.024)	<0.0001		1.005	(0.995, 1.015)	0.348
Prevalence of diabetes (QOF 2006/07)	1.108	(1.020, 1.203)	0.015		1.095	(1.008, 1.187)	0.031
Percentage over 65	1.060	(1.038, 1.083)	<0.0001		1.067	(1.044, 1.091)	<0.0001
Percentage male patients	1.073	(1.035, 1.111)	<0.0001		1.058	(1.021, 1.097)	0.002
Number of GPs per 1000 patients	1.209	(0.894, 1.637)	0.218		1.113	(0.821, 1.508)	0.491
Hypertension detection 2006/07 (QOF 2006/07)	0.984	(0.955, 1.014)	0.300		0.988	(0.959, 1.018)	0.416
% patients offered smoking cessation advice (SM02 - QOF 2006/07)	1.006	(0.996, 1.016)	0.271		1.010	(1.000, 1.021)	0.057
% serum cholesterol (CHD08 - QOF 2006/07)	0.989	(0.980, 0.999)	0.028		0.992	(0.983, 1.002)	0.109
% aspirin (CHD09 - QOF 2006/07)	1.007	(0.986, 1.029)	0.514		1.003	(0.982, 1.025)	0.777
% of patients with recalled perception of being able to see preferred GP (QOF 2006/07)	0.995	(0.990, 1.000)	0.069		0.995	(0.990, 1.000)	0.061
%smoking prevalence – estimated (QOF 2006/07)					1.031	(1.012, 1.052)	0.002

[1]IRR, 95% confidence intervals and associated p values as a result of negative binomial model of count of premature mortality caused by CHD.

For peer review only

Competing Interests

KH had financial support from CLAHRC in the form of funding for PhD fees. RB is in receipt of an NIHR Senior Investigator award. No financial relationships with any organisations that might have an interest in the submitted work in the previous five years; no other relationships or activities that could appear to have influenced the submitted work.

Authors' contributions

Contributor Statement

The study was conceived by KH, RB, JB and DJ. KH designed the study, carried out the analysis and drafted the initial manuscript. JB and DJ contributed to the statistical analysis. RB, JB and DJ contributed to drafting and editing the final manuscript and interpreting and reviewing the results of the statistical analysis.

Acknowledgements

The research was funded and led by National Institute for Health Research (NIHR) Collaboration for Leadership in Applied Health Research and Care) based at LNR. RB is in receipt of an NIHR Senior Investigator award. The views expressed are those of the authors and not necessarily those of the NHS, the NIHR or the Department of Health. The funders had no role in the study design, data collection and analysis, decision to publish or preparation of the manuscript.

Ethical committee approval

NRES advised that NHS ethics committee was not required.

For peer review only

Bibliography

[1] Brown J. *Smoking prevalence in England below 20% for the first time in 80 years;* 2014 http://www.bmj.com/content/348/bmj.f7535/rr/683849 (accessed 3 Feb 2014)

[2] Corgan E. *Local Stop Smoking Services: Service deliver and monitoring guidance*; 2011 https://www.gov.uk/-government/uploads/system/uploads/attachment_data/file/213755/dh_125939.pdf (accessed 3 Feb 2014)

[3] Clinical Commissioning Strategy (Updated April 2013) 2012-2015; 2013. https://www.leicestercityccg.nhs.uk/-wp-content/uploads/2013/09/Commissioning-Strategy-v20.1.pdf (accessed 3 Feb 2014)

[4] Overview of CHD Programme 2013-2014;. Accessed on 8 Feb 2014. Available from: http://-www.cambridgeshireandpeterboroughccg.nhs.uk/coronary-heart-disease.htm (accessed 3 Feb 2014)

[5] Soljak M, Calderon-Larrañaga A, Sharma P, Cecil E, Bell D, Abi-Aad G, et al. *Does higher quality primary health care reduce stroke admissions? A national cross-sectional study.* Br J Gen Pract. 2011;61(593):e801–e807.

[6] Purdy S, Griffin T, Salisbury C *et al*. *Emergency admissions for coronary heart disease: a cross-sectional study of general practice, population and hospital factors in England.* Public Health. 2011;125(1):46–54.

[7] Bankart MJG, Baker R, Rashid A *et al*. *Characteristics of general practices associated with emergency admission rates to hospital: a cross-sectional study.* Emerg Med J. 2011 Jul;28(7):558–563.

[8] Kiran T, Hutchings A, Dhalla IA *et al*. *The association between cardiovascular outcomes: a cross-sectional study using data from the UK Quality and Outcomes Framework.* J Epidemiol Community Health. 2010;64:927–934.

[9] Honeyford K, Baker R, Bankart MJG *et al*. *Modelling factors in primary care quality improvement: a cross-sectional study of premature CHD mortality.* BMJ Open. 2013;3(10):e003391. doi:10.1136/bmjopen-2013-003391

[10] Twigg L, Moon G, Walker S. *The smoking epidemic in England*. London: Health Development Agency; 2004. http://www.nice.org.uk/niceMedia/documents/smoking_epidemic.pdf (accessed 10 Feb 2014).

[11] Bryant G, Chappel D, Unsworth L. *The Prevalence of Smoking in the North East - Occasional Paper No. 49*. North East Public Health Observatory; 2012.

[12] Office for National Statistics *Sample Design and Response - Appendix B - General Lifestyle Survey*. Office for National Statistics; 2011. http://www.ons.gov.uk/ons/rel/ghs/general-lifestyle-survey/2011/index.html (accessed 10 Feb 2014)

[13] Office for National Statistics. *Statistical Bulletin - Integrated Household Survey April 2010 to March 2011: Experimental Statistics.* Office for National Statistics; 2011.

[14] Blak BT, Thompson M, Dattani H, Bourke A. *Generalisability of The Health Improvement Network (THIN) database: demographics, chronic disease prevalence and mortality rates*. Inform Prim Care. 2011;19(4):251–255.

[15] QRESEARCH. The QRESEARCH database. http://www.qresearch.org/SitePages/Home.aspx (accessed 1 Feb 2014)

[16] Szatkowski L, Lewis S, McNeill A *et al*. *Can data from primary care medical records be used to monitor national smoking prevalence?* J Epidemiol Community Health. 2012;66(9):791–795.

[17] Simpson CR, Hippisley-Cox J and Sheikh A. *Trends in the epidemiology of smoking recorded in UK general practice.* British Journal of General Practice. 2010;60(572):121–127.

[18] Health and Social Care Information Centre. *Annex A: Quality indicators - Summary of points*. Health and Social Care Information Centre; 2004. http://www.hscic.gov.uk/catalogue/PUB01946 (accessed 1 Feb 2014)

[19] Health and Social Care Information Centre. *Frequently Asked Questions* http://qof.hscic.gov.uk/faqs/-index.asp#qof23 (accessed 1 Feb 2014)

[20] Health and Social Care Information Centre. *Quality and Outcomes Framework* http://www.hscic.gov.uk/qof (accessed 1 Feb 2014).

[21] London Health Observatory. *Smoking prevalence among adults aged 18+ by region and local authority*. Updated August 2012 http://www.lho.org.uk/viewResource.aspx?id=16678 (accessed 3 Dec 2013)

[22] Bland JM and Altman DG. *Statistical methods for assessing agreement between two methods of clinical measurement*. Lancet. 1986;1(8476):307–310.

[23] Lin LI. *A concordance correlation coefficient to evaluate reproducibility*. Biometrics. 1989;45(1):255–268.

[24] Doran T, Kontopantelis E, Fullwood C *et al*. *Exempting dissenting patients from pay for performance schemes: retrospective analysis of exception reporting in the UK Quality and Outcomes Framework*. BMJ. 2012;344:e2405–e2405.

[25] Wong S, Shields M, Leatherdale S *et al*. *Assessment of validity of self-reported smoking status*. Health Rep. 2012;23(1):47–53.

For peer review only

[26] Shipton D, Tappin DM, Vadiveloo T *et al*. *Reliability of self reported smoking status by pregnant women for estimating smoking prevalence: a retrospective, cross sectional study*. BMJ. 2009;339:b4347–b4347.
[27] Strong M, Maheswaran R, Pearson T. *A comparison of methods for calculating general practice level socioeconomic deprivation*. Int J Health Geogr. 2006;5(1):29.
[28] McLean G, Guthrie B, Watt G *et al*. *Practice postcode versus patient population: a comparison of data sources in England and Scotland*. Int J Health Geogr. 2008;7(1):37.
[29] Brettell R, Soljak M, Cecil E *et al*. *Reducing heart failure admission rates in England 2004-2011 are not related to changes in primary care quality: national observational study*. European Journal of Heart Failure. 2013;15(12):1335-1342 doi: 10.1093/eurjhf/hft107.
[30] Coleman T. *Do financial incentives for delivering health promotion counselling work? Analysis of smoking cessation activities stimulated by the quality and outcomes framework*. BMC public health. 2010;10(1):167.
[31] Zwar NA, Mendelsohn CP and Richmond RL *Supporting smoking cessation.* BMJ. 2014;348:f7535
[32] Kontopantelis E, Springate D, Reeves D *et al*. *Withdrawing performance indicators: retrospective analysis of general practice performance under UK Quality and Outcomes Framework*. BMJ. 2014;348:g330. Available from: http://-dx.doi.org/10.1136/bmj.g330.

For peer review only

Figure 1 Relationship between aggregated QOF estimates and IHS estimates for local authority districts

Legend Fig 1a Association between estimates (dashed line: estimates are equal)

Fig 1b Bland-Altman plot showing relationship between difference in estimates and mean difference (solid line: mean difference; dashed lines: 95% limits of agreement)

QOF estimates based on 2012/13 data; IHS estimates based on 2011/12 survey.

Figure 2 Relationship between QOF estimates for the general population and those with chronic conditions (2012/13)

Legend Fig 2a Association between estimates (dashed line: estimates are equal; solid line: fitted line)

Fig 2b Bland-Altman plot showing relationship between difference in estimates and mean difference (solid line: mean difference; dashed lines: 95% limits of agreement)

SM07 and SM08 (2012/13) used for QOF estimates for the general population;

SM05 and SM06 (2012/13) used for QOF estimates for those with chronic conditions.

For peer review only

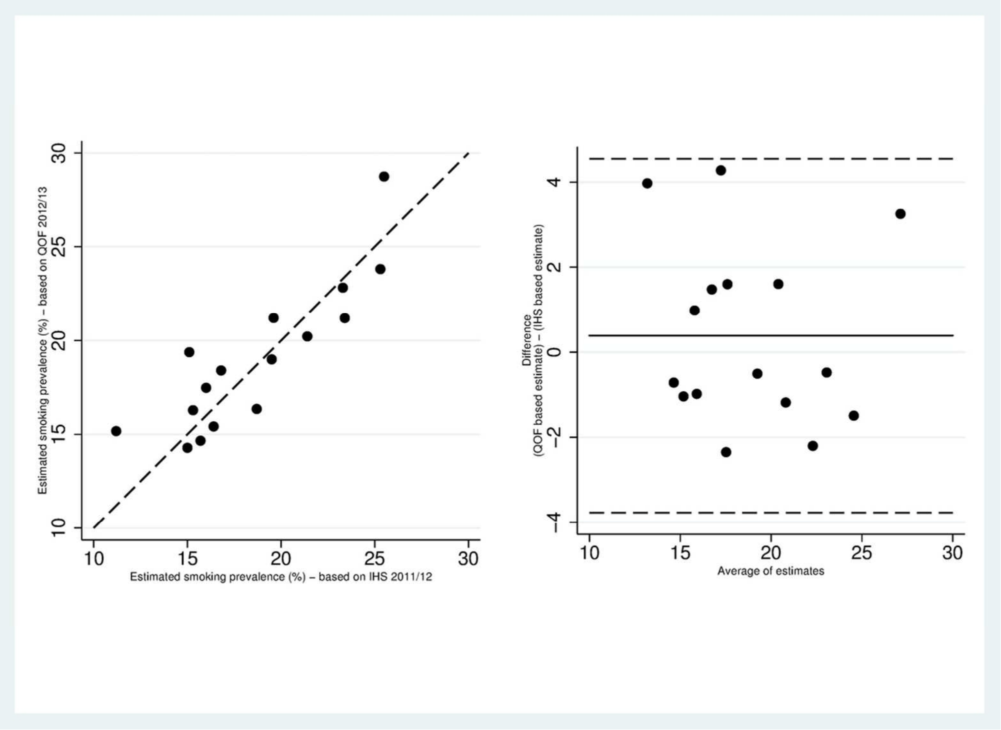

Figure 1 Relationship between aggregated QOF estimates and IHS estimates for local authority districts
Legend Fig 1a Association between estimates (dashed line: estimates are equal)
Fig 1b Bland-Altman plot showing relationship between difference in estimates and mean difference (solid line: mean difference; dashed lines: 95% limits of agreement)
QOF estimates based on 2012/13 data; IHS estimates based on 2011/12 survey.

101x73mm (300 x 300 DPI)

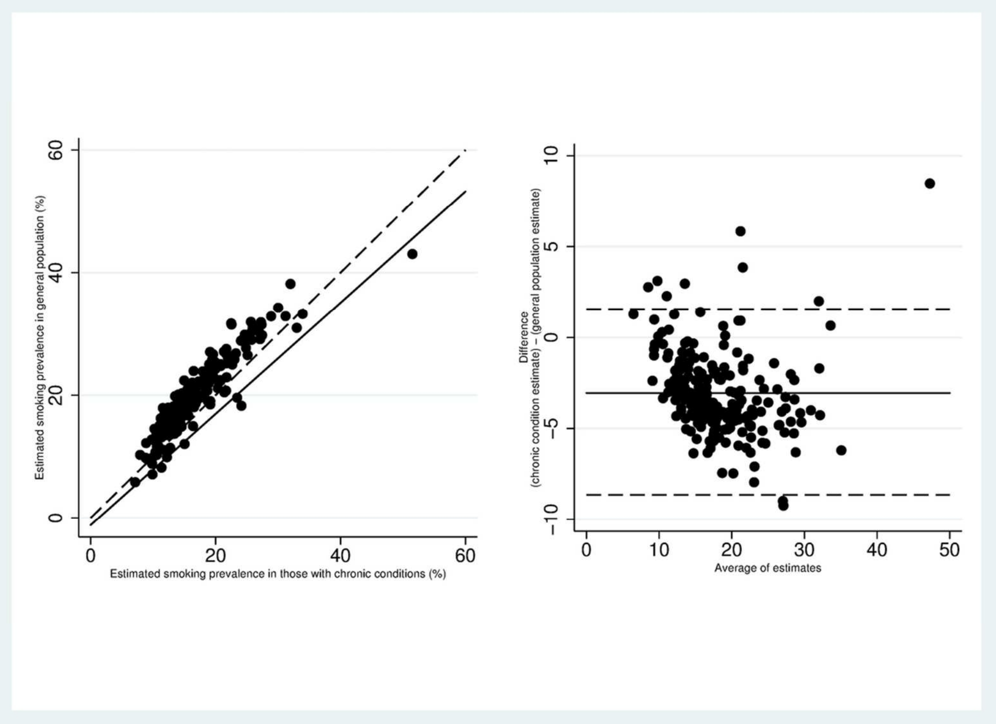

Figure 2 Relationship between QOF estimates for the general population and those with chronic conditions (2012/13)
Legend Fig 2a Association between estimates (dashed line: estimates are equal; solid line: fitted line)
Fig 2b Bland-Altman plot showing relationship between difference in estimates and mean difference (solid line: mean difference; dashed lines: 95% limits of agreement)
SM07 and SM08 (2012/13) used for QOF estimates for the general population;
SM05 and SM06 (2012/13) used for QOF estimates for those with chronic conditions.

101x73mm (300 x 300 DPI)

only

Table S1 Concordance between estimates of smoking prevalence in those with chronic conditions 2006/07 to 2012/13

Year	2012/13	2011/12	2010/11	2009/10	2008/09	2007/08
2011/12	0.97<br>*-0.15[1] (-2.4, 2.7)[2]*					
2010/11	0.97<br>*-0.12 (-2.8, 2.6)*	0.97<br>*0.04 (-2.6, 2.7)*				
2009/10	0.96<br>*-0.12 (-3.3, 3.0)*	0.96<br>*-0.03 (-3.2, 3.2)*	0.99<br>*0.00 (-2.0, 2.0)*			
2008/09	0.95<br>*0.06 (-3.5, 3.7)*	0.95)<br>*0.22 (-3.4, 3.8)*	0.97<br>*0.18 (-2.5, 2.9)*	0.98<br>*0.19 (-1.9, 2.3)*		
2007/08	0.93<br>*0.71 (-3.0, 4.6)*	0.93<br>*0.87 (-3.0, 4.8)*	0.95<br>*0.83 (-2.4, 4.0)*	0.96<br>*0.84 (-1.8, 3.5)*	0.97<br>*0.65 (-1.74, 3.0)*	
2006/07	0.93<br>*0.64 (-3.6, 4.6)*	0.92<br>*0.79 (-3.4, 5.0)*	0.94<br>*0.76 (-2.8, 4.4)*	0.94<br>*0.76 (-2.6, 4.1)*	0.95<br>*0.57 (-2.9, 4.1)*	0.97<br>*-0.08 (-2.6, 2.5)*

Lin's concordance coefficients
$p<0.001$ for all coefficients
Mean difference[1] and 95% Limits of Agreement[2] are given in italics

For peer review only

STROBE 2007 (v4) Statement—Checklist of items that should be included in reports of *cross-sectional studies*

Section/Topic	Item #	Recommendation	Reported on page #
**Title and abstract**	1	(*a*) Indicate the study's design with a commonly used term in the title or the abstract	3 - abstract
		(*b*) Provide in the abstract an informative and balanced summary of what was done and what was found	3 - abstract
**Introduction**			
Background/rationale	2	Explain the scientific background and rationale for the investigation being reported	4 - background
Objectives	3	State specific objectives, including any prespecified hypotheses	3 - abstract<br>4 - background
**Methods**			
Study design	4	Present key elements of study design early in the paper	3 -abstract
Setting	5	Describe the setting, locations, and relevant dates, including periods of recruitment, exposure, follow-up, and data collection	5 – methods – 'sample'
Participants	6	(*a*) Give the eligibility criteria, and the sources and methods of selection of participants	5 – methods – 'sample'
Variables	7	Clearly define all outcomes, exposures, predictors, potential confounders, and effect modifiers. Give diagnostic criteria, if applicable	5 - methods
Data sources/ measurement	8*	For each variable of interest, give sources of data and details of methods of assessment (measurement). Describe comparability of assessment methods if there is more than one group	5 - methods
Bias	9	Describe any efforts to address potential sources of bias	5 – methods & 6 – results (recording of smoking status and prevalence)
Study size	10	Explain how the study size was arrived at	5 – methods – 'sample'
Quantitative variables	11	Explain how quantitative variables were handled in the analyses. If applicable, describe which groupings were chosen and why	5 - methods

For peer review only

Statistical methods	12	(*a*) Describe all statistical methods, including those used to control for confounding	5 – methods
		(*b*) Describe any methods used to examine subgroups and interactions	5 – methods
		(*c*) Explain how missing data were addressed	5 – methods
		(*d*) If applicable, describe analytical methods taking account of sampling strategy	5 – methods
		(*e*) Describe any sensitivity analyses	6 - results
**Results**			
Participants	13*	(a) Report numbers of individuals at each stage of study—eg numbers potentially eligible, examined for eligibility, confirmed eligible, included in the study, completing follow-up, and analysed	5 - methods
		(b) Give reasons for non-participation at each stage	
		(c) Consider use of a flow diagram	
Descriptive data	14*	(a) Give characteristics of study participants (eg demographic, clinical, social) and information on exposures and potential confounders	5 - methods
		(b) Indicate number of participants with missing data for each variable of interest	
Outcome data	15*	Report numbers of outcome events or summary measures	6 - results
Main results	16	(*a*) Give unadjusted estimates and, if applicable, confounder-adjusted estimates and their precision (eg, 95% confidence interval). Make clear which confounders were adjusted for and why they were included	
		(*b*) Report category boundaries when continuous variables were categorized	n/a
		(*c*) If relevant, consider translating estimates of relative risk into absolute risk for a meaningful time period	n/a
Other analyses	17	Report other analyses done—eg analyses of subgroups and interactions, and sensitivity analyses	5 – methods<br>6 - results
**Discussion**			
Key results	18	Summarise key results with reference to study objectives	7 – discussion – principal findings
Limitations	19	Discuss limitations of the study, taking into account sources of potential bias or imprecision. Discuss both direction and magnitude of any potential bias	7 – discussion – strengths and weaknesses
Interpretation	20	Give a cautious overall interpretation of results considering objectives, limitations, multiplicity of analyses, results from similar studies, and other relevant evidence	7 - discussion
Generalisability	21	Discuss the generalisability (external validity) of the study results	7 - discussion
**Other information**			

For peer review only

Funding	22	Give the source of funding and the role of the funders for the present study and, if applicable, for the original study on which the present article is based	11 – competing interests

*Give information separately for cases and controls in case-control studies and, if applicable, for exposed and unexposed groups in cohort and cross-sectional studies.

Note: An Explanation and Elaboration article discusses each checklist item and gives methodological background and published examples of transparent reporting. The STROBE checklist is best used in conjunction with this article (freely available on the Web sites of PLoS Medicine at http://www.plosmedicine.org/, Annals of Internal Medicine at http://www.annals.org/, and Epidemiology at http://www.epidem.com/). Information on the STROBE Initiative is available at www.strobe-statement.org.

For peer review only

BMJ Open

Estimating smoking prevalence in general practice using data from the Quality and Outcomes Framework (QOF)

Journal:	*BMJ Open*
Manuscript ID:	bmjopen-2014-005217.R1
Article Type:	Research
Date Submitted by the Author:	10-Jun-2014
Complete List of Authors:	Honeyford, Kate; University of Leicester, Health Sciences<br>Baker, Richard; University of Leicester, Health Sciences<br>Bankart, M. John; Keele University, Institute of Primary Care and Healthe Sciences<br>Jones, David; University of Leicester, Health Sciences
**Primary Subject Heading**:	Epidemiology
Secondary Subject Heading:	Health services research, Public health, Smoking and tobacco
Keywords:	EPIDEMIOLOGY, STATISTICS & RESEARCH METHODS, PUBLIC HEALTH, PRIMARY CARE

SCHOLARONE™
Manuscripts

For peer review only

TITLE

Estimating smoking prevalence in general practice using data from the Quality and Outcomes Framework (QOF)

AUTHORS' INFORMATION

Corresponding author:
Kate Honeyford MSc
Adrian Building,
University Road,
Leicester
LE1 7RH
ceh28@le.ac.uk
Tel: 0116 229 7254/7255
Fax: 0116 229 7250
Co-authors:
Professor Richard Baker, Dept of Health Sciences, University of Leicester, Leicester, UK.
Dr M. John G. Bankart, Institute of Primary Care and Health Sciences, Keele University, Keele, UK.
Professor David R Jones, Dept of Health Sciences, University of Leicester, Leicester, UK.

KEYWORDS
Smoking/epidemiology
Population surveillance
Primary Health Care
Cardiovascular disease

WORD COUNT
3422 words

For peer review only

Objectives

To determine to what extent underlying data published as part of QOF can be used to estimate smoking prevalence within practice populations and local areas and to explore the usefulness of these estimates.

Design

Cross-sectional, observational study of QOF smoking data. Smoking prevalence in general practice populations and among patients with chronic conditions was estimated by simple manipulation of QOF indicator data. Agreement between estimates from the integrated household survey (IHS) and aggregated QOF-based estimates were calculated. The impact of including smoking estimates in negative binomial regression models of counts of premature CHD deaths was assessed.

Setting

Primary care in the East Midlands.

Participants

All general practices in the area of study were eligible for inclusion (230). 14 practices were excluded due to incomplete QOF data for the period of study (2006/07 – 2012/13). One practice was excluded as it served a restricted practice list.

Measurements

Estimates of smoking prevalence in general practice populations and among patients with chronic conditions.

Results

Median smoking prevalence in the practice populations for 2012/13 was 19.2% (range 5.8% - 43.0%). There was good agreement (mean difference: 0.39%; 95% limits of agreement (-3.77, 4.55)) between IHS estimates for local authority districts and aggregated QOF register estimates. Smoking prevalence estimates in those with chronic conditions were lower than for the general population (mean difference -3.05%), but strongly correlated (R_p=0.74, p<0.0001). An important positive association between premature CHD mortality and smoking prevalence was shown when smoking prevalence was added to other population and service characteristics.

Conclusions

Published QOF data allow useful estimation of smoking prevalence within practice populations and in those with chronic conditions; the latter estimates may sometimes be useful in place of the former. It may also provide useful estimates of smoking prevalence in local areas by aggregating practice based data.

For peer review only

Strengths and limitations of this study

- This paper clearly demonstrates that useful estimations of smoking prevalence within practice populations can be calculated from routine data published through the Quality and Outcomes Framework (QOF).
- Our analysis shows that estimates of smoking prevalence in those with chronic conditions can be used in some situations in place of an estimate for the general population, if this is not available.
- Comparisons with local area estimates suggest QOF-based estimates are useful for estimating smoking prevalence in both practice populations and in local areas.
- QOF data rely on self-reported smoking status, recorded in the previous 27 months, which may underestimate smoking status or the effectiveness of interventions.
- This study does not have access to individual patient data limiting our understanding of patients who do not have smoking status recorded and the possible impact of missing data on estimates of smoking prevalence.

For peer review only

Background

Despite smoking prevalence in England falling "below 20% for the first time in 80 years",[1] reducing smoking remains a key public health priority in England as in many countries, with local authorities and primary care services being expected to play a key role in local tobacco control services.[2] In addition, clinical commissioning groups (CCGs), membership organisations responsible for planning, organising and purchasing nationally funded healthcare within their local areas have their own health targets. Reducing smoking prevalence is a key component of many targets, for example reducing chronic obstructive pulmonary disease (COPD) outcomes [3] and reducing inequalities in coronary heart disease (CHD).[4] Reliable estimates of smoking prevalence for practice populations and local areas are useful to assess need, inform targeting of interventions delivered through primary care and to evaluate those interventions. For practices and Clinical Commissioning Groups (CCGs) it is important to be able to evaluate different approaches to smoking cessation and to understand the different level of risk in different practices. Practice based estimates are of particular importance for research into a variety of health outcomes and their associations with primary care. Research of this type generally aims to take characteristics of the practice populations into account and the inclusion of smoking prevalence has been shown to be important in the interpretation of other factors, in particular socio-economic deprivation [5-6] Currently a variety of measures of smoking prevalence in practice populations are being used [6-7] and some studies do not include a measure of smoking,[8-10] despite the recognized associations between smoking prevalence and a range of chronic conditions.[11]

National Surveys

In England, there are various national surveys of smoking prevalence including the Health Survey for England;[12] the General Lifestyle Survey;[13] the Smoking Toolkit Study (STS) and the Integrated Household Survey (IHS). The IHS began in 2009 and is a composite survey including questions on smoking habits (involving over 420,000 adults in 2011). IHS statistics are designated as experimental, in a 'testing phase' and not yet fully developed,[14] but estimates are available for local authorities. It could, therefore, be argued that there is no gold standard measure of smoking in local areas, and there are no surveys which aim to establish the smoking prevalence within practice populations.

Patient records

Analyses of individual patient records, using the THIN (The Health Improvement Network) [15] and QRESEARCH [16] databases, provide strong evidence that smoking status within primary care medical records could be used to monitor national smoking patterns. There was good agreement between smoking prevalence based on medical records in the THIN database and those predicted by GHS; 22.4% compared to 21.8% respectively in males; 18.9% compared to 20.2% respectively in females.[17] Estimates of smoking prevalence based on the medical records in the QRESEARCH database has also shown good agreement with national surveys, in this case the Health Survey for England.[18]

QOF

The national Quality and Outcomes Framework (QOF) is a payment for performance system which was introduced in England in 2004 to improve the quality of primary care for patients. Practices are awarded points for achieving targets and these points are translated into financial reward. Since its inception QOF has included indicators relating to smoking.[19] The underlying aim of these indicators has not changed over the years; a) practices should record smoking status in patient notes and, b) for those who smoke, smoking cessation advice/support/treatment should have been offered. Until 2012/13 the focus was on targeting smoking cessation advice to those with chronic conditions. Table 1 summarises QOF smoking indicators 2006/7 – 2014/15.

The QOF indicators have not been designed to determine smoking prevalence within the practice population; indeed it is clearly stated that 'QOF provides no information on numbers of smokers and non-smokers',[20] attributing this mainly to the condition-specific nature of the indicator. The wording has not changed since the inclusion of the two new indicators which apply to the general population and are not condition-specific.

Objective

In this paper we aim to explore to what extent underlying data published as part of QOF can be used to estimate smoking prevalence within practice populations. The usefulness of these estimates are explored by (i) comparing aggregated data with local area estimates from other sources and (ii) including practice level estimates in a model of CHD mortality.

For peer review only

For peer review only

METHOD

Sample

All practices within three primary care trusts (PCTs), the organizational unit for administering general practices in England, (2006/07 to 2011/12) in the East Midlands were eligible for inclusion in the study. 215 practices with QOF data available for the seven financial years were included in the analysis. 14 practices were excluded because they lacked data for all seven years. One practice was excluded from the study as it served a restricted practice list; another practice was excluded from the study as 2012/13 QOF data strongly suggested an error.

Manipulation of QOF data

QOF data can be downloaded from the Health and Social Care Information Centre website containing information for all practices in a region;[22] Table 2 illustrates the type of data available.
Two key QOF indicators are used in the calculations of smoking prevalence in the total practice population:

- SM07 ‘The percentage of patients aged 15 years and over whose notes record smoking status in the preceding 27 months’
- SM08 ‘The percentage of patients aged 15 years and over who are recorded as current smokers who have a record of an offer of support and treatment within the preceding 27 months’

These can be summarized as follows:

SMOKING STATUS INDICATOR (SM07) =

$$No.\,of\ patients\ who\ have\ their\ smoking\ status\ recorded \Big/ No.\,of\ eligible\ patients\ in\ the\ practice$$

SMOKING CESSATION INDICATOR (SM08) =

$$No.\,of\ patients\ who\ have\ a\ record\ of\ cessation\ support \Big/ No.\,of\ patients\ recorded\ as\ current\ smokers$$

The denominator of the SMOKING STATUS INDICATOR (SM07) provides an estimate of the sample of the practice population whose smoking status should be recorded. This includes the whole practice population aged over 15, with the exception of people who have joined the practice in the three months prior to the data extraction point and patients who refuse to provide their smoking status.
The denominator of the SMOKING CESSATION INDICATOR (SM08) provides an estimate of those who are recorded as current smokers.
In addition indicators of a similar nature were included but applying to those with any, or any combination, of a range of QOF specified chronic conditions (SM05 and SM06).

For peer review only

Using the data given for these indicators, it is possible to estimate the smoking prevalence in a practice population, summarised below.

SMOKING PREVALENCE ESTIMATE =

$$\text{No. of patients recorded as current smokers}\Big/\text{No. of eligible patients in the practice}$$

$$= \text{Denominator of } SM08 \Big/ \text{Denominator of } SM07$$

For example, for practice A the denominator for SM07 is 3721 – the number of eligible patients in the practice. The denominator for SM08 is 1129 - indicating that there are 1129 registered patients recorded as current smokers. Hence smoking prevalence can be estimated as 1129/3721 or 30.3%. Table 2 gives worked examples for five practices. This method was used to estimate smoking prevalence for the total practice population in 2013/14 and, using appropriate indicators, for those with chronic conditions from 2006/07 to 2013/14 (SM05 and SMO06 in 2013/14).

In addition, the percentage of the practice population with a chronic condition was determined using the denominator of SM07 as a measure of the practice population and the denominator of SM05 as a measure of the practice population with a chronic condition.

Comparisons with local area estimates

Practice postcodes were linked to local authority districts using the National Statistics Postcode Directory (NSPD) [23] and then confirmed by visual check of addresses. Practice level data were aggregated, to estimate smoking prevalence in local authority districts. Details of the estimated population of each district, the aggregated population for which smoking status has been determined, the number of practices in each district and the sample size for the IHS 2011/12 are included in Table 3.

These estimates were compared to estimates of smoking prevalence in local authority districts based on data from the Integrated Household Survey.[24]

Modelling

To determine the importance of being able to estimate smoking prevalence in practice populations, the estimate of smoking prevalence was included in a model to determine the associations of premature CHD (under 75) mortality with various population and service characteristics; the methods are described by Honeyford et al.[10] Here, counts of premature CHD deaths (between April 2006 and March 2009) were modelled using negative binomial regression, using the same explanatory variables but including estimated smoking prevalence for those with chronic conditions based on QOF 2006/07. Service and population characteristics derived from QOF registers from 2006/07 were originally selected for inclusion in the study but an estimate of smoking prevalence for the general population was not available for this year.

For peer review only

RESULTS

Estimates using QOF data

Estimation of overall smoking prevalence using QOF smoking indicators 2012/13

The median underlying achievement for the recording of smoking status in the total practice population was 88.1% in 2012/13 (IQR: (83.7, 91.0)). The median estimate of smoking prevalence in practice populations was 19.2%, ranging from 5.8% to 43.0% (IQR: (15.1%, 22.9%)).

Estimates of smoking prevalence in those with chronic conditions using QOF smoking indicators 2012/13

The underlying achievement for recording smoking status in those with chronic conditions was higher than for the total practices population (96.6% IQR (95.0, 97.7)). The median practice based estimate for those with any or any combination of a specific list of chronic conditions was 15.4% (IQR: 12.6% to 19.4%), ranging from 7.1% to 51.5%.

The estimates of smoking prevalence in those with chronic conditions have been consistent since 2006/07, with the median varying slightly during that time. Concordance was high between estimates for all years; Lin's concordance coefficient [25] was greater than 0.92 and mean difference was less than one in all cases (Table S1 in Appendix for more details).

Comparisons with local area estimates

Estimates of smoking prevalence were in line with estimates derived from the IHS. Aggregating over the total area, smoking prevalence was 19.5%, compared to 19.3% when IHS district level data were aggregated over the same area.

When practice data were combined to give estimates of smoking for local authority districts there was a strong positive correlation (Rp=0.86, p<0.0001) and good agreement (mean difference: 0.39%; 95% limits of agreement (-3.77, 4.55)) between estimates based on QOF registers and IHS estimates (Fig. 1).[26]

When the estimates of prevalence for those with chronic conditions were aggregated into local authority districts, estimates were lower than IHS estimates for the majority of areas.

Associations between measures

Association between smoking prevalence in the general practice population and those with chronic conditions.

Smoking prevalence in those with chronic conditions was lower than in the general practice population. The mean difference between the two estimates was -3.05% (95% limits of agreement: (-8.65, 1.56)).The Bland-Altman plot does not suggest a strong pattern, despite some evidence that the difference increases as the average increases (Fig. 2).

There was a strong positive correlation (R_p=0.92, p<0.0001) between the overall estimate of smoking prevalence within a practice population and in those with chronic conditions. A regression model was developed to predict smoking prevalence in the general population based on the prevalence in those with chronic conditions; removal of outliers improved model fit.

Associations between recording of smoking status and prevalence

There was a strong positive correlation between recording of smoking status in the general population and in those with chronic conditions (underlying achievement for SM07 and SM05 respectively) (R_p=0.74, p<0.0001). There was no evidence of an association between smoking prevalence in the general population and recording of smoking status (R_p=-0.07, p=0.28) or the percentage with a chronic condition (R_p=0.03, p=0.67).

Including smoking prevalence estimates in models of mortality

Table 4 shows incident rate ratios, 95% CIs and associated p values for the original and modified models. Inclusion of the smoking prevalence variable in the model reduced the strength of the associations between deprivation and premature mortality, and percentage white and premature mortality. Sensitivity analysis considering the impact of exception reporting indicates no impact on interpretation (see Doran [27] for details of exception reporting).

DISCUSSION

Principal findings

These results show how the QOF registers required as part of the general practice pay for performance scheme in England can be used to estimate smoking prevalence in practice populations and that these estimates are useful when analysing patterns of mortality. Practice based estimates can be aggregated to provide estimates of smoking prevalence in local areas.

When smoking prevalence is estimated in the general population using QOF indicators there is good agreement with estimates of IHS smoking prevalence for similar geographical areas.

For peer review only

QOF data can also be used to estimate smoking prevalence in those with chronic conditions, which is generally lower than smoking prevalence in the general population. There is good agreement between the estimates in successive years. The correlation between estimates of smoking prevalence in the general population in 2012/13 and those with chronic conditions is strong. These strong correlations suggest that the estimates based on previous years can be used in place of smoking prevalence in the general population for some purposes. Regression analysis suggests that smoking prevalence in those with chronic conditions can be used to predict smoking prevalence in the general practice population, for practices with a typical patient list.

When an estimate of smoking prevalence in those with chronic conditions was used in a study of the association between premature CHD mortality and various population and service characteristics an important positive association between CHD mortality and smoking prevalence was shown.

Strengths and weaknesses

The agreement between IHS based area estimates of smoking prevalence and estimates based on combining QOF data provides evidence to suggest that manipulating QOF data results is a useful measure of smoking prevalence within practice populations when compared to other available measures. This is supported by the work of Szatkowski et al [17] which found good agreement between national smoking prevalence predicted by patient records and the General Household Survey. In addition, practice based QOF data can be aggregated to provide local area estimates of smoking prevalence based on a much larger sample size than other surveys.

When comparing practices and analysing patterns across practices, it is important that the estimate is consistent across practices. The percentage of patients who do not have their smoking status recorded varies from 40% to less than 1%, but the characteristics of these patients are not known. Recording of smoking status has been shown to vary between groups [18-19, 28]; women, older people and those with chronic conditions were more likely to have their smoking status recorded. National surveys suggest that smoking rates are lower in these groups and therefore smoking prevalence from QOF may underestimate actual smoking prevalence. The implications of this will vary between practices, dependent on the proportion of these groups within their practice populations. Our analysis did not find an association between the percentage with a chronic condition and the recording of smoking status in the total population or the estimate of smoking prevalence.

QOF data are based on self-reported smoking status, which has been shown to be reliable in the general population,[28] but to underestimate smoking prevalence in pregnant women.[29] In addition, practices are only asked to record smoking status in the preceding 27 months, meaning the estimates may be useful in assessing need and analysing associations, but will have disadvantages in assessing the effectiveness of interventions, unless practices commit to more regular recording.

Practice level smoking data have been aggregated to local authority districts based on practice postcode rather than patient postcodes. General practice catchments are not constrained by local authority boundaries, however studies have shown that 80% of patients live within a 10 minute car journey of their practice [30], suggesting that patients choose practices close to where they live. It is relatively common for practice postcodes to be used as a proxy for patient postcodes; however, when used to estimate deprivation this has been found to underestimate relationships between deprivation and health outcomes.[31-32]

Further work using individual patient records is necessary to analyse the frequency of recording of smoking status and the characteristics of patients for whom no smoking status is recorded or have been excluded on the basis of exception reporting. In this analysis practice level data have been aggregated to estimate smoking prevalence in local authority districts. Analysis of patient level postcode information, not available for this study, would allow estimates of smoking prevalence for smaller geographical areas to be made. These could then be compared to modelled estimates or locally commissioned surveys, where they exist.

Implications

Manipulating QOF data is an easy and cost effective method of estimating smoking prevalence in both practice populations and local areas, although further work is necessary to determine the validity of using aggregated practice level data for local area estimation. Both local area and practice based estimates are important to those tasked with reducing smoking rates and improving the nation's health. CCGs and public health departments in local authorities need them to target smoking cessation and other additional resources. Understanding more about the patient populations

For peer review only

would enable similar practices to be compared when considering differences in health outcomes and the apparent effectiveness of interventions.[33]

Current estimates of smoking prevalence in local areas are based on the Integrated Household Study. The IHS is currently in an experimental phase since the weighting methodology needs to be assessed and potentially revised.[34] Aggregated practice level data includes the majority of the resident adult population in local areas and could therefore be a more useful measure of local area smoking prevalence, at district level and at smaller local areas than are currently available through the IHS. Analysis of patient level geographical data is necessary to determine the potential utility of simple and more complex aggregation methods.

When estimates of smoking prevalence are included in the analysis of the associations between premature CHD mortality and practice population and service characteristics, there are reductions in the magnitude of the incident rate ratios (IRRs) for both deprivation and percentage white. This suggests that these may be acting as surrogate markers of other lifestyle factors, such as smoking prevalence. Hence, the lack of reliable smoking information may be leading to relative over emphasis being placed on socio-economic deprivation, often described using an index of multiple factors. Reliable measures of smoking prevalence will improve our understanding of the relative importance of deprivation and other characteristics in explaining inequalities in a variety of health outcomes.

Smoking prevalence in those with chronic conditions is typically lower than in the general population. This may be due to diagnosis increasing motivation to quit smoking,[35] the increase in smoking cessation advice and support [36] or the age and gender profile of those with chronic conditions. Smoking prevalence in those with chronic conditions has not reduced over the seven year period covered in this analysis, possibly suggesting that smoking cessation advice has limited effect, but this may be due to the turnover of patients with chronic conditions as a result of both premature mortality and new diagnoses. A wide range of smoking cessation advice and support has recently been reviewed by Zwar et al;[37] consideration of how these impact on those with chronic conditions is recommended as a result of this finding.

QOF smoking indicators have changed since 2004 and continue to change. The introduction, in 2012/13, of an indicator which allows estimates of the smoking prevalence within the general population is useful for researchers as well as CCGs and public health officials. The removal of the indicator that covers the recording of smoking status in the total population from QOF in 2014/15 will impact on the methodology described in this paper, although the number of patients who are recorded as current smokers will continue to be available. The population of the practice will need to be used as the denominator in the calculation of smoking prevalence. It will be important to determine if the smoking status declines after the removal of the indicator; a recent study suggests that removal of indicators does not lead to a decline in clinical activities.[38]

Conclusion

Data published through QOF allow useful estimations of smoking prevalence within practice populations and in those with chronic conditions to be made. These estimates are important in developing our understanding of differences in health outcomes between practices, and are useful to both individual practices and CCGs when comparing practice level health outcomes, to assess need and to inform targeting. Aggregating practice level data may also be useful to allow estimates of smoking prevalence in local areas to be made. Revisions to QOF means that researchers will need to update methodology as indicators change.

For peer review only

Table 1 Summary of smoking indicators for which underlying achievement is published

General form of the indicator	Patient group	
	Patients with any, or any combination of the following conditions: coronary heart disease, stroke or TIA, hypertension, diabetes, COPD or asthma[1].	All patients aged 15 years+
**% of patients whose notes record smoking status[2]**	SM01: 2006/07 & 2007/08<br>SM03: 2008/09 – 2011/12<br>SM05: 2012/13<br>SMOK002: 2013/14 – 2014/15	Records 22: 2006/07 & 2007/08<br>Records 23: 2008/09 – 2011/12<br>SM07: 2012/13<br>SMOK001: 2013/14 – retired in 2014/15
**% of patients who are recorded as current smokers whose notes contain a record that smoking cessation advice or referral to a specialist service, where available, has been offered within the previous 15 months[3]**	SM02: 2006/07 & 2007/08<br>SM04: 2008/09 – 2011/12<br>SM06: 2012/13<br>SMOK005: 2013/14 – 2014/15	SM08: 2012/13<br>SMOK004: 2013/14 – 2014/15
**The practice supports smokers in stopping smoking by a strategy which includes providing literature and offering appropriate therapy.**		Information 5: 2006/07-2011/12<br>SMOK003: 2012/13 – 2014/15

[1]In 2008/09 CKD, asthma, schizophrenia, bipolar affective disorder or other psychoses were added to the list of chronic conditions and in 2012/13 PAD was added.
[2]For those with chronic conditions, the record must have been made in the past 15 months, reduced to 12 months in 2013/14, for all patients the period is 27 months, reduced to 24 months in 2013/14.
[3]In 2012/13 this changed to 'who have a record of an offer of support and treatment within the preceding 15 months', the period is 27 months for all patients, reduced to 12 months and 24 months respectively in 2013/14.

For peer review only

Table 2 Example of QOF data from 2012/13, showing how it can be used to calculate smoking prevalence for individual practices.

		Example practices				
QOF description	Interpretation for purposes of calculating smoking prevalence	A	B	C	D	E
SM07 Points		11	10.5	10.8	9.6	11
SM07 Numerator	Patients[1] whose notes contain a record of smoking status	3450	1319	6276	31948	6504
SM07 Denominator	Patients who are eligible to be included in this indicator[2]	3721	1497	7033	37654	7212
SM07 UA		92.70%	88.10%	89.20%	84.80%	90.20%
SM08 Points		12	9.9	12	8.9	12
SM08 Numerator	Patients who are recorded as current smokers and have a record of an offer of support etc	1024	325	1578	8439	2165
SM08 Denominator	Patients who are recorded as current smokers	1129	401	1586	10931	2373
SM08 UA		90.70%	81.00%	99.50%	77.20%	91.20%
	Calculation to determine percentage who are smokers SM08 den/ SM07 den	1129/3721	401/1497	1586/7033	10931/37654	2373/7212
	Estimate of smoking prevalence	30.30%	26.80%	22.60%	29.00%	32.90%

1[1]*Patients aged over 15*

[2]*For example patients who are newly registered with the practices (less than three months) are excluded from the indicator*

For peer review only

Table 3: Comparison of the population of each district based on the 2011 Census and aggregation QOF based practice data.

**Local authority**	Population aged 15 and over (2011 Census)[1]	Population included in QOF indicator SM07[2]	Number of general practices[3]	IHS sample size 2011/12[4]
Leicestershire				
Blaby	77600	67895	9	301
Charnwood	139800	152533	24	396
Harborough	70200	69168	8	234
Hinckley and Bosworth	87800	84159	12	305
Melton	41900	34912	2	130
North West Leicestershire	77000	78331	14	242
Oadby and Wigston	47100	48054	9	167
**Northamptonshire**				
Corby	49400	57112	5	131
Daventry	64100	71902	8	223
East Northamptonshire	70900	55279	8	217
Kettering	75900	87059	9	180
Northampton	171600	184370	27	446
South Northamptonshire	69700	60391	8	205
Wellingborough	61300	61013	9	172
**Unitary Authorities**				
Leicester	264600	293156	59	1475
Rutland	31300	29628	4	416
Totals	1400200	1434962	215	5240

[1] Data based on 2011 Census available from ONS [21]
[2] Based on QOF registers accessed from [22]
[3] Practices are matched to local authority districts based on the postcode of the practice [23].
[4] Based on IHS data 2011/12 [24]

For peer review only

Table 4 Estimated incident rate ratios (IRRs) for premature (U75) CHD mortality count (n=215)[1].

	***without** smoking prevalence variable*				***with** smoking prevalence variable*		
**Explanatory variable**	***IRR***	***95% CI***	***p value***		***IRR***	***95% CI***	***p value***
Percentage white patients	1.007	(1.003, 1.012)	0.002		1.001	(0.995, 1.007)	0.657
Deprivation score (IMD 2007)	1.017	(1.011, 1.024)	<0.0001		1.005	(0.995, 1.015)	0.348
Prevalence of diabetes (QOF 2006/07)	1.108	(1.020, 1.203)	0.015		1.095	(1.008, 1.187)	0.031
Percentage over 65	1.060	(1.038, 1.083)	<0.0001		1.067	(1.044, 1.091)	<0.0001
Percentage male patients	1.073	(1.035, 1.111)	<0.0001		1.058	(1.021, 1.097)	0.002
Number of GPs per 1000 patients	1.209	(0.894, 1.637)	0.218		1.113	(0.821, 1.508)	0.491
Hypertension detection 2006/07 (QOF 2006/07)	0.984	(0.955, 1.014)	0.300		0.988	(0.959, 1.018)	0.416
% patients offered smoking cessation advice (SM02 - QOF 2006/07)	1.006	(0.996, 1.016)	0.271		1.010	(1.000, 1.021)	0.057
% serum cholesterol (CHD08 - QOF 2006/07)	0.989	(0.980, 0.999)	0.028		0.992	(0.983, 1.002)	0.109
% aspirin (CHD09 - QOF 2006/07)	1.007	(0.986, 1.029)	0.514		1.003	(0.982, 1.025)	0.777
% of patients with recalled perception of being able to see preferred GP (QOF 2006/07)	0.995	(0.990, 1.000)	0.069		0.995	(0.990, 1.000)	0.061
%smoking prevalence – estimated (QOF 2006/07)					1.031	(1.012, 1.052)	0.002

[1]IRR, 95% confidence intervals and associated p values as a result of negative binomial model of count of premature mortality caused by CHD.

For peer review only

Acknowledgements

The research was funded and led by National Institute for Health Research (NIHR) Collaboration for Leadership in Applied Health Research and Care) based at LNR. RB is in receipt of an NIHR Senior Investigator award. The views expressed are those of the authors and not necessarily those of the NHS, the NIHR or the Department of Health. The funders had no role in the study design, data collection and analysis, decision to publish or preparation of the manuscript.

Competing Interests

KH had financial support from CLAHRC in the form of funding for PhD fees. RB is in receipt of an NIHR Senior Investigator award. No financial relationships with any organisations that might have an interest in the submitted work in the previous five years; no other relationships or activities that could appear to have influenced the submitted work.

Authors' contributions

Contributor Statement

The study was conceived by KH, RB, JB and DJ. KH designed the study, carried out the analysis and drafted the initial manuscript. JB and DJ contributed to the statistical analysis. RB, JB and DJ contributed to drafting and editing the final manuscript and interpreting and reviewing the results of the analysis.

Ethical committee approval

NRES advised that NHS ethics committee was not required.

Data sharing

No additional data is available

For peer review only

Bibliography

[1] Brown J. *Smoking prevalence in England below 20% for the first time in 80 years;* 2014 http://www.bmj.com/content/348/bmj.f7535/rr/683849 (accessed 3 Feb 2014)
[2] Corgan E. *Local Stop Smoking Services: Service deliver and monitoring guidance*; 2011 https://www.gov.uk/-government/uploads/system/uploads/attachment_data/file/213755/dh_125939.pdf (accessed 3 Feb 2014)
[3] Clinical Commissioning Strategy (Updated April 2013) 2012-2015; 2013. https://www.leicestercityccg.nhs.uk/-wp-content/uploads/2013/09/Commissioning-Strategy-v20.1.pdf (accessed 3 Feb 2014)
[4] Overview of CHD Programme 2013-2014;. Accessed on 8 Feb 2014. Available from: http://-www.cambridgeshireandpeterboroughccg.nhs.uk/coronary-heart-disease.htm (accessed 3 Feb 2014)
[5] Brettell R, Soljak M, Cecil E *et al*. *Reducing heart failure admission rates in England 2004-2011 are not related to changes in primary care quality: national observational study*. European Journal of Heart Failure. 2013;15(12):1335-1342 doi: 10.1093/eurjhf/hft107.
[6] Purdy S, Griffin T, Salisbury C *et al*. *Emergency admissions for coronary heart disease: a cross-sectional study of general practice, population and hospital factors in England.* Public Health. 2011;125(1):46–54.
[7] Soljak M, Calderon-Larrañaga A, Sharma P, et al. *Does higher quality primary health care reduce stroke admissions? A national cross-sectional study.* Br J Gen Pract. 2011;61(593):e801–e807.
[8] Bankart MJG, Baker R, Rashid A *et al*. *Characteristics of general practices associated with emergency admission rates to hospital: a cross-sectional study.* Emerg Med J. 2011 Jul;28(7):558–563.
[9] Kiran T, Hutchings A, Dhalla IA *et al*. *The association between cardiovascular outcomes: a cross-sectional study using data from the UK Quality and Outcomes Framework.* J Epidemiol Community Health. 2010;64:927–934.
[10] Honeyford K, Baker R, Bankart MJG *et al*. *Modelling factors in primary care quality improvement: a cross-sectional study of premature CHD mortality.* BMJ Open. 2013;3(10):e003391. doi:10.1136/bmjopen-2013-003391
[11] Twigg L, Moon G, Walker S. *The smoking epidemic in England*. London: Health Development Agency; 2004. http://www.nice.org.uk/niceMedia/documents/smoking_epidemic.pdf (accessed 10 Feb 2014).
[12] Bryant G, Chappel D, Unsworth L. *The Prevalence of Smoking in the North East - Occasional Paper No. 49*. North East Public Health Observatory; 2012.
[13] Office for National Statistics *Sample Design and Response - Appendix B - General Lifestyle Survey*. Office for National Statistics; 2011. http://www.ons.gov.uk/ons/rel/ghs/general-lifestyle-survey/2011/index.html (accessed 10 Feb 2014)
[14] Office for National Statistics. *Statistical Bulletin - Integrated Household Survey April 2010 to March 2011: Experimental Statistics.* Office for National Statistics; 2011.
[15] Blak BT, Thompson M, Dattani H, Bourke A. *Generalisability of The Health Improvement Network (THIN) database: demographics, chronic disease prevalence and mortality rates*. Inform Prim Care. 2011;19(4):251–255.
[16] QRESEARCH. The QRESEARCH database. http://www.qresearch.org/SitePages/Home.aspx (accessed 1 Feb 2014)
[17] Szatkowski L, Lewis S, McNeill A *et al*. *Can data from primary care medical records be used to monitor national smoking prevalence?* J Epidemiol Community Health. 2012;66(9):791–795.
[18] Simpson CR, Hippisley-Cox J and Sheikh A. *Trends in the epidemiology of smoking recorded in UK general practice.* British Journal of General Practice. 2010;60(572):121–127.
[19] Health and Social Care Information Centre. *Annex A: Quality indicators - Summary of points*. Health and Social Care Information Centre; 2004. http://www.hscic.gov.uk/catalogue/PUB01946 (accessed 1 Feb 2014)
[20] Health and Social Care Information Centre. *Frequently Asked Questions* http://qof.hscic.gov.uk/faqs/-index.asp#qof23 (accessed 1 Feb 2014)
[21] Office for National Statistics *Table P04 2011 Census: Usual resident population by five-year age group, local authorities in England and Wales.* 2012 http://www.ons.gov.uk/ons/publications/re-reference-tables.html?edition=tcm%3A77-257414 (accessed 3 June 2014)
[22] Health and Social Care Information Centre. *Quality and Outcomes Framework* http://www.hscic.gov.uk/qof (accessed 1 Feb 2014).
[23] Office for National Statistics *National Statistics Postcode Products* http://www.ons.gov.uk/ons/guide-method/geography/products/postcode-directories/-nspp-/index.html (accessed 3 June 2014)
[24] London Health Observatory. *Smoking prevalence among adults aged 18+ by region and local authority*. Updated August 2012 http://www.lho.org.uk/viewResource.aspx?id=16678 (accessed 3 Dec 2013)

For peer review only

[25] Lin LI. *A concordance correlation coefficient to evaluate reproducibility*. Biometrics. 1989;45(1):255–268.

[26] Bland JM and Altman DG. *Statistical methods for assessing agreement between two methods of clinical measurement*. Lancet. 1986;1(8476):307–310.

[27] Doran T, Kontopantelis E, Fullwood C *et al*. *Exempting dissenting patients from pay for performance schemes: retrospective analysis of exception reporting in the UK Quality and Outcomes Framework*. BMJ. 2012;344:e2405–e2405.

[28] Wong S, Shields M, Leatherdale S *et al*. *Assessment of validity of self-reported smoking status*. Health Rep. 2012;23(1):47–53.

[29] Haynes R, Lovett, A and Sünnerberg G *Potential accessibility, travel time, and consumer choice: geographical variations in general medical practice registrations in Eastern England* Environment and Panning A 2003 35(10):1733-1750

[30] Shipton D, Tappin DM, Vadiveloo T *et al*. *Reliability of self reported smoking status by pregnant women for estimating smoking prevalence: a retrospective, cross sectional study*. BMJ. 2009;339:b4347–b4347.

[31] Strong M, Maheswaran R, Pearson T. *A comparison of methods for calculating general practice level socioeconomic deprivation*. Int J Health Geogr. 2006;5(1):29.

[32] McLean G, Guthrie B, Watt G *et al*. *Practice postcode versus patient population: a comparison of data sources in England and Scotland*. Int J Health Geogr. 2008;7(1):37.

[33] Sullivan E, Baker R, Jones D *et al Primary healthcare teams' views on using mortality to review clinical policies.* Qual Saf Health Care. 2007;16:359-362

[34] Office for National Statistics: Statistical Bulletin – Integrated Household Survey April 2010 to March 2011: Experimental Statistics 2011 http://www.ons.gov.uk/ons/dcp171778_227150.pdf (accessed 3rd June 2014)

[35] Bassett JC, Gore JL, Chi, AC *et al. Impact of bladder cancer diagnosis on smoking behaviour.* J Clin Oncol 2012; 30:1871-1878

[36] Coleman T. *Do financial incentives for delivering health promotion counselling work? Analysis of smoking cessation activities stimulated by the quality and outcomes framework*. BMC public health. 2010;10(1):167.

[37] Zwar NA, Mendelsohn CP and Richmond RL *Supporting smoking cessation.* BMJ. 2014;348:f7535

[38] Kontopantelis E, Springate D, Reeves D *et al*. *Withdrawing performance indicators: retrospective analysis of general practice performance under UK Quality and Outcomes Framework*. BMJ. 2014;348:g330. Available from: http://-dx.doi.org/10.1136/bmj.g330.

For peer review only

Figure 1 Relationship between aggregated QOF estimates and IHS estimates for local authority districts

Legend Fig 1a Association between estimates (dashed line: estimates are equal)

Fig 1b Bland-Altman plot showing relationship between difference in estimates and mean difference (solid line: mean difference; dashed lines: 95% limits of agreement)

QOF estimates based on 2012/13 data; IHS estimates based on 2011/12 survey.

Figure 2 Relationship between QOF estimates for the general population and those with chronic conditions (2012/13)

Legend Fig 2a Association between estimates (dashed line: estimates are equal; solid line: fitted line)

Fig 2b Bland-Altman plot showing relationship between difference in estimates and mean difference (solid line: mean difference; dashed lines: 95% limits of agreement)

SM07 and SM08 (2012/13) used for QOF estimates for the general population;

SM05 and SM06 (2012/13) used for QOF estimates for those with chronic conditions.

For peer review only

TITLE

~~Estimating smoking prevalence in general practices: an evaluation of QOF (Quality and Outcomes Framework) data~~

Estimating smoking prevalence in general practice using data from the Quality and Outcomes Framework (QOF)

AUTHORS' INFORMATION

Corresponding author:
Kate Honeyford MSc
Adrian Building,
University Road,
Leicester
LE1 7RH
ceh28@le.ac.uk
Tel: 0116 229 7254/7255
Fax: 0116 229 7250
Co-authors:
Professor Richard Baker, Dept of Health Sciences, University of Leicester, Leicester, UK.
Dr M. John G. Bankart, Institute of Primary Care and Health Sciences, Keele University, Keele, UK.
Professor David R Jones, Dept of Health Sciences, University of Leicester, Leicester, UK.

KEYWORDS
Smoking/epidemiology
Population surveillance
Primary Health Care
Cardiovascular disease

WORD COUNT

~~2987~~ 3422 words

For peer review only

Objectives
To determine to what extent underlying data published as part of QOF can be used to estimate smoking prevalence within practice populations and local areas and to explore the usefulness of these estimates.

Design
Cross-sectional, observational study of QOF smoking data. Smoking prevalence in general practice populations and among patients with chronic conditions was estimated by simple manipulation of QOF indicator data. Agreement between estimates from the integrated household survey (IHS) and aggregated QOF-based estimates were calculated. The impact of including smoking estimates in negative binomial regression models of counts of premature CHD deaths was assessed.

Setting
Primary care in the East Midlands.

Participants
All general practices in the area of study were eligible for inclusion (230). 14 practices were excluded due to incomplete QOF data for the period of study (2006/07 – 2012/13). One practice was excluded as it served a restricted practice list.

Measurements
Estimates of smoking prevalence in general practice populations and among patients with chronic conditions.

Results
Median smoking prevalence in the practice populations for 2012/13 was 19.2% (range 5.8% - 43.0%). There was good agreement (mean difference: 0.39%; 95% limits of agreement (-3.77, 4.55)) between IHS estimates for local authority districts and aggregated QOF register estimates. Smoking prevalence estimates in those with chronic conditions were lower than for the general population (mean difference -3.05%), but strongly correlated (R_p=0.74, p<0.0001). An important positive association between premature CHD mortality and smoking prevalence was shown when smoking prevalence was added to other population and service characteristics.

Conclusions
Published QOF data allow useful estimation of smoking prevalence within practice populations and in those with chronic conditions; the latter estimates may sometimes be useful in place of the former. It may also provide useful estimates of smoking prevalence in local areas by aggregating practice based data.

For peer review only - http://bmjopen.bmj.com/site/about/guidelines.xhtml

Strengths and limitations of this study

- This paper clearly demonstrates that useful estimations of smoking prevalence within practice populations can be calculated from routine data published through the Quality and Outcomes Framework (QOF).
- Our analysis shows that estimates of smoking prevalence in those with chronic conditions can be used in some situations in place of an estimate for the general population, if this is not available.
- Comparisons with local area estimates suggest QOF-based estimates are useful for estimating smoking prevalence in both practice populations and in local areas.
- QOF data rely on self-reported smoking status, recorded in the previous 27 months, which may underestimate smoking status or the effectiveness of interventions.
- This study does not have access to individual patient data limiting our understanding of patients who do not have smoking status recorded and the possible impact of missing data on estimates of smoking prevalence.

For peer review only

Background

Despite smoking prevalence in England falling "below 20% for the first time in 80 years",[1] reducing smoking remains a key public health priority in England as in many countries, with local authorities and primary care services being expected to play a key role in local tobacco control services.[2] In addition, clinical commissioning groups (CCGs), membership organisations responsible for planning, organising and purchasing nationally funded healthcare within their local areas have their own health targets. Reducing smoking prevalence is a key component of many targets, for example reducing chronic obstructive pulmonary disease (COPD) outcomes [3] and reducing inequalities in coronary heart disease (CHD).[4] ~~Access to reliable estimates of smoking prevalence in practice populations is useful to assess need, inform targeting of interventions delivered through primary care and to evaluate those interventions. In addition, research into a variety of health outcomes and their associations with primary care needs to take characteristics of the practice populations into account. Currently a variety of measures of smoking prevalence in practice populations are being used [5-6] and some do not include smoking,[7-9] despite the recognized associations between smoking prevalence and a range of chronic conditions.[10]~~ Reliable estimates of smoking prevalence for practice populations and local areas are useful to assess need, inform targeting of interventions delivered through primary care and to evaluate those interventions. For practices and Clinical Commissioning Groups (CCGs) it is important to be able to evaluate different approaches to smoking cessation and to understand the different level of risk in different practices. Practice based estimates are of particular importance for research into a variety of health outcomes and their associations with primary care. Research of this type generally aims to take characteristics of the practice populations into account and the inclusion of smoking prevalence has been shown to be important in the interpretation of other factors, in particular socio-economic deprivation [5-6] Currently a variety of measures of smoking prevalence in practice populations are being used [6-7] and some studies do not include a measure of smoking,[8-10] despite the recognized associations between smoking prevalence and a range of chronic conditions.[11]

National Surveys

In England, there are various national surveys of smoking prevalence including the Health Survey for England;[12] the General Lifestyle Survey;[13] the Smoking Toolkit Study (STS) and the Integrated Household Survey (IHS). The IHS began in 2009~~;~~ and is a composite survey including questions on smoking habits (involving over 420,000 adults in 2011). IHS statistics are designated as experimental, in a 'testing phase' and not yet fully developed,[14] but estimates are available for local authorities. ~~None of these surveys aims to establish the smoking prevalence within practice populations.~~ It could, therefore, be argued that there is no gold standard measure of smoking in local areas, and there are no surveys which aim to establish the smoking prevalence within practice populations.

Patient records

Analyses of individual patient records, using the THIN (The Health Improvement Network) [15] and QRESEARCH [16] databases, provide strong evidence that smoking status within primary care medical records could be used to monitor national smoking patterns. There was good agreement between smoking prevalence based on medical records in the THIN database and those predicted by GHS; 22.4% compared to 21.8% respectively in males; 18.9% compared to 20.2% respectively in females.[17] Estimates of smoking prevalence based on the medical records in the QRESEARCH database has also shown good agreement with national surveys, in this case the Health Survey for England.[18]

QOF

~~The national Quality and Outcomes Framework (QOF) was introduced in England in 2004 to improve the quality of primary care for patients.~~The national Quality and Outcomes Framework (QOF) is a payment for performance system which was introduced in England in 2004 to improve the quality of primary care for patients. Practices are awarded points for achieving targets and these points are translated into financial reward. Since its inception QOF has included indicators relating to smoking.[19] The underlying aim of these indicators has not changed over the years; a) practices should record smoking status in patient notes and, b) for those who smoke, smoking cessation advice/support/treatment should have been offered. Until 2012/13 the focus was on targeting smoking cessation advice to those with chronic conditions. Table 1 summarises QOF smoking indicators 2006/7 – 2014/15.

The QOF indicators have not been designed to determine smoking prevalence within the practice population; indeed it is clearly stated that 'QOF provides no information on numbers of smokers and non-smokers',[20] attributing this mainly to the condition-specific nature of the indicator. The wording has not changed since the inclusion of the two new

For peer review only

indicators which apply to the general population and are not condition-specific.

Objective

~~In this paper we aim to explore to what extent underlying data published as part of QOF can be used to estimate smoking prevalence within practice populations and to evaluate the usefulness of such estimates.~~

In this paper we aim to explore to what extent underlying data published as part of QOF can be used to estimate smoking prevalence within practice populations. The usefulness of these estimates are explored by (i) comparing aggregated data with local area estimates from other sources and (ii) including practice level estimates in a model of CHD mortality.

For peer review only

METHOD

Sample

All practices within three primary care trusts (PCTs), the organizational unit for administering general practices in England, (2006/07 to 2011/12) in the East Midlands were eligible for inclusion in the study. 215 practices with QOF data available for the seven financial years were included in the analysis. 14 practices were excluded because they lacked data for all seven years. One practice was excluded from the study as it served a restricted practice list; another practice was excluded from the study as 2012/13 QOF data strongly suggested an error.

Manipulation of QOF data

QOF data can be downloaded from the Health and Social Care Information Centre website containing information for all practices in a region;[22] Table 2 illustrates the type of data available.

Two key QOF indicators are used in the calculations of smoking prevalence in the total practice population:

- SM07 'The percentage of patients aged 15 years and over whose notes record smoking status in the preceding 27 months'
- SM08 'The percentage of patients aged 15 years and over who are recorded as current smokers who have a record of an offer of support and treatment within the preceding 27 months'

These can be summarized as follows:

SMOKING STATUS INDICATOR (SM07) =

$$\textit{No. of patients who have their smoking status recorded} \Big/ \textit{No. of eligible patients in the practice}$$

SMOKING CESSATION INDICATOR (SM08) =

$$\textit{No. of patients who have a record of cessation support} \Big/ \textit{No. of patients recorded as current smokers}$$

The denominator of the SMOKING STATUS INDICATOR (SM07) provides an estimate of the sample of the practice population whose smoking status should be recorded. This includes the whole practice population aged over 15, with the exception of people who have joined the practice in the three months prior to the data extraction point and patients who refuse to provide their smoking status.

The denominator of the SMOKING CESSATION INDICATOR (SM08) provides an estimate of those who are recorded as current smokers.

In addition indicators of a similar nature were included but applying to those with any, or any combination, of a range of QOF specified chronic conditions (SM05 and SM06).

For peer review only

Using the data given for these indicators, it is possible to estimate the smoking prevalence in a practice population, summarised below.

SMOKING PREVALENCE ESTIMATE =

$$\text{No. of patients recorded as current smokers} \big/ \text{No. of eligible patients in the practice}$$

$$= \text{Denominator of } SM08 \big/ \text{Denominator of } SM07$$

For example, for practice A the denominator for SM07 is 3721 – the number of eligible patients in the practice. The denominator for SM08 is 1129 - indicating that there are 1129 registered patients recorded as current smokers. Hence smoking prevalence can be estimated as 1129/3721 or 30.3%. Table 2 gives worked examples for five practices. This method was used to estimate smoking prevalence for the total practice population in 2013/14 and, using appropriate indicators, for those with chronic conditions from 2006/07 to 2013/14 (SM05 and SMO06 in 2013/14).

In addition, the percentage of the practice population with a chronic condition was determined using the denominator of SM07 as a measure of the practice population and the denominator of SM05 as a measure of the practice population with a chronic condition.

Comparisons with local area estimates

Practice postcodes were linked to local authority districts using the National Statistics Postcode Directory (NSPD) [23] and then confirmed by visual check of addresses. Practice level data were aggregated, to estimate smoking prevalence in local authority districts. Details of the estimated population of each district, the aggregated population for which smoking status has been determined, the number of practices in each district and the sample size for the IHS 2011/12 are included in Table 3.

These estimates were compared to estimates of smoking prevalence in local authority districts based on data from the Integrated Household Survey.[24]

Modelling

To determine the importance of being able to estimate smoking prevalence in practice populations, the estimate of smoking prevalence was included in a model to determine the associations of premature CHD (under 75) mortality with various population and service characteristics; the methods are described by Honeyford et al.[10] Here, counts of premature CHD deaths (between April 2006 and March 2009) were modelled using negative binomial regression, using the same explanatory variables but including estimated smoking prevalence for those with chronic conditions based on QOF 2006/07. Service and population characteristics derived from QOF registers from 2006/07 were originally selected for inclusion in the study but an estimate of smoking prevalence for the general population was not available for this year.

RESULTS

Estimates using QOF data

Estimation of overall smoking prevalence using QOF smoking indicators 2012/13

The median underlying achievement for the recording of smoking status in the total practice population was 88.1% in 2012/13 (IQR: (83.7, 91.0)). The median estimate of smoking prevalence in practice populations was 19.2%, ranging from 5.8% to 43.0% (IQR: (15.1%, 22.9%)).

Estimates of smoking prevalence in those with chronic conditions using QOF smoking indicators 2012/13

The underlying achievement for recording smoking status in those with chronic conditions was higher than for the total practices population (96.6% IQR (95.0, 97.7)). The median practice based estimate for those with any or any combination of a specific list of chronic conditions was 15.4% (IQR: 12.6% to 19.4%), ranging from 7.1% to 51.5%. The estimates of smoking prevalence in those with chronic conditions have been consistent since 2006/07, with the median varying slightly during that time. Concordance was high between estimates for all years; Lin's concordance coefficient [25] was greater than 0.92 and mean difference was less than one in all cases (Table S1 in Appendix for more details).

Comparisons with local area estimates

Estimates of smoking prevalence were in line with estimates derived from the IHS. Aggregating over the total area, smoking prevalence was 19.5%, compared to 19.3% when IHS district level data were aggregated over the same area. When practice data were combined to give estimates of smoking for local authority districts there was a strong positive correlation (Rp=0.86, p<0.0001) and good agreement (mean difference: 0.39%; 95% limits of agreement (-3.77, 4.55)) between estimates based on QOF registers and IHS estimates (Fig. 1).[26]

When the estimates of prevalence for those with chronic conditions were aggregated into local authority districts, estimates were lower than IHS estimates for the majority of areas.

Associations between measures

Association between smoking prevalence in the general practice population and those with chronic conditions.

Smoking prevalence in those with chronic conditions ~~is~~ was lower than in the general practice population. The mean difference between the two estimates was -3.05% (95% limits of agreement: (-8.65, 1.56)).The Bland-Altman plot does not suggest a strong pattern, despite some evidence that the difference increases as the average increases (Fig. 2). There was a strong positive correlation (R_p=0.92, p<0.0001) between the overall estimate of smoking prevalence within a practice population and in those with chronic conditions. A regression model was developed to predict smoking prevalence in the general population based on the prevalence in those with chronic conditions; removal of outliers improved model fit.

Associations between recording of smoking status and prevalence

There was a strong positive correlation between recording of smoking status in the general population and in those with chronic conditions (underlying achievement for SM07 and SM05 respectively) (R_p=0.74, p<0.0001). There was no evidence of an association between smoking prevalence in the general population and recording of smoking status (R_p=-0.07, p=0.28) or the percentage with a chronic condition (R_p=0.03, p=0.67).

~~**QOF smoking indicators 2006/07-2012/13**~~

~~The estimates of smoking prevalence in those with chronic conditions have been consistent since 2006/07, with the median varying slightly during that time. Concordance was high between estimates for all years; Lin's concordance coefficient 3] was greater than 0.92 and mean difference was less than one in all cases (Table S1 in Appendix for more details).~~

Including smoking prevalence estimates in models of mortality

Table 4~~3~~ shows incident rate ratios, 95% CIs and associated *p* values for the original and modified models. Inclusion of the smoking prevalence variable in the model reduced the strength of the associations between deprivation and premature mortality, and percentage white and premature mortality. ~~A one unit increase in smoking prevalence was associated with an increase of 3.2% in expected premature CHD mortality count. If a practice with a moderately high smoking prevalence (75th percentile: 18.86%) is compared to one with a median level of smoking prevalence (15.09%), a difference of 11.69% in premature CHD mortality count can be expected, after adjusting for the other variables in the model.~~

Sensitivity analysis considering the impact of exception reporting indicates no impact on interpretation (see Doran [27] for details of exception reporting).

Discussion

Principal findings

These results show how the QOF registers required as part of the general practice pay for performance scheme in England can be used to estimate smoking prevalence in practice populations and that these estimates are useful when analysing patterns of mortality. Practice based estimates can be aggregated to provide estimates of smoking prevalence in local areas.

~~These results show how QOF registers can be used to estimate smoking prevalence in practice populations and that these estimates are useful when analysing patterns of mortality.~~

When smoking prevalence is estimated in the general population using QOF indicators there is good agreement with estimates of IHS smoking prevalence for similar geographical areas.

QOF data can also be used to estimate smoking prevalence in those with chronic conditions, which is generally lower than smoking prevalence in the general population. There is good agreement between the estimates in successive years. The correlation between estimates of smoking prevalence in the general population in 2012/13 and those with chronic conditions is strong. These strong correlations suggest that the estimates based on previous years can be used in place of smoking prevalence in the general population for some purposes. Regression analysis suggests that smoking prevalence in those with chronic conditions can be used to predict smoking prevalence in the general practice population, for practices with a typical patient list.

When an estimate of smoking prevalence in those with chronic conditions was used in a study of the association between premature CHD mortality and various population and service characteristics an important positive association between CHD mortality and smoking prevalence was shown.

Strengths and weaknesses

The agreement between IHS based area estimates of smoking prevalence and estimates based on combining QOF data provides evidence to suggest that manipulating QOF data results is a useful measure of smoking prevalence within practice populations when compared to other available measures. This is supported by the work of Szatkowski et al [17] which found good agreement between national smoking prevalence predicted by patient records and the General Household Survey. In addition, practice based QOF data can be aggregated to provide local area estimates of smoking prevalence based on a much larger sample size than other surveys.

When comparing practices and analysing patterns across practices, it is important that the estimate is consistent across practices. The percentage of patients who do not have their smoking status recorded varies from 40% to less than 1%, but the characteristics of these patients are not known. Recording of smoking status has been shown to vary between groups [18-19, 28]; women, older people and those with chronic conditions were more likely to have their smoking status recorded. National surveys suggest that smoking rates are lower in these groups and therefore smoking prevalence from QOF may underestimate actual smoking prevalence. The implications of this will vary between practices, dependent on the proportion of these groups within their practice populations. Our analysis did not find an association between the percentage with a chronic condition and the recording of smoking status in the total population or the estimate of smoking prevalence.

QOF data are based on self-reported smoking status, which has been shown to be reliable in the general population,[28] but to underestimate smoking prevalence in pregnant women.[29] In addition, practices are only asked to record smoking status in the preceding 27 months, meaning the estimates may be useful in assessing need and analysing associations, but will have disadvantages in assessing the effectiveness of interventions, unless practices commit to more regular recording.

~~Practice level data have been aggregated to local authority districts based on practice postcode rather than patient postcodes; it is relatively common for practice postcodes to be used as a proxy for patient postcodes but when used to estimate deprivation has been found to underestimate relationships between deprivation and health outcomes. Further work using individual patient records is necessary to analyse the frequency of recording of smoking status and the characteristics of patients for whom no smoking status is recorded or have been excluded on the basis of exception reporting.~~

Practice level smoking data have been aggregated to local authority districts based on practice postcode rather than patient postcodes. General practice catchments are not constrained by local authority boundaries, however studies have shown that 80% of patients live within a 10 minute car journey of their practice [30], suggesting that patients

choose practices close to where they live. It is relatively common for practice postcodes to be used as a proxy for patient postcodes; however, when used to estimate deprivation this has been found to underestimate relationships between deprivation and health outcomes.[31-32]
Further work using individual patient records is necessary to analyse the frequency of recording of smoking status and the characteristics of patients for whom no smoking status is recorded or have been excluded on the basis of exception reporting. In this analysis practice level data have been aggregated to estimate smoking prevalence in local authority districts. Analysis of patient level postcode information, not available for this study, would allow estimates of smoking prevalence for smaller geographical areas to be made. These could then be compared to modelled estimates or locally commissioned surveys, where they exist.

Implications

Having estimates of the smoking prevalence in practice populations is important to those Manipulating QOF data is an easy and cost effective method of estimating smoking prevalence in both practice populations and local areas, although further work is necessary to determine the validity of using aggregated practice level data for local area estimation. Both local area and practice based estimates are important to those -tasked with reducing smoking rates and improving the nation's health. CCGs and public health departments in local authorities need them to target smoking cessation and other additional resources. Understanding more about the patient populations would enable similar practices to be compared when considering differences in health outcomes and the apparent effectiveness of interventions.[33]

Current estimates of smoking prevalence in local areas are based on the Integrated Household Study. The IHS is currently in an experimental phase since the weighting methodology needs to be assessed and potentially revised.[34] Aggregated practice level data includes the majority of the resident adult population in local areas and could therefore be a more useful measure of local area smoking prevalence, at district level and at smaller local areas than are currently available through the IHS. Analysis of patient level geographical data is necessary to determine the potential utility of simple and more complex aggregation methods.

When estimates of smoking prevalence are included in the analysis of the associations between premature CHD mortality and practice population and service characteristics, there are reductions in the magnitude of the incident rate ratios (IRRs) for both deprivation and percentage white. This suggests that these may be acting as surrogate markers of other lifestyle factors, such as smoking prevalence. Hence, the lack of reliable smoking information may be leading to relative over emphasis being placed on socio-economic deprivation, often described using an index of multiple factors. Reliable measures of smoking prevalence will improve our understanding of the relative importance of deprivation and other characteristics in explaining inequalities in a variety of health outcomes.
Similarly, it was found that social class was not linked to hospital admissions for stroke and CHD when rates were adjusted for various factors including smoking.[27] However, even with smoking prevalence included in the models, Brettell et al [29] found that increased deprivation was associated with higher heart failure admission rates, and Purdy et al [6] found that higher deprivation was associated with increased emergency admissions for myocardial infarction and angina. Unless we have reliable measures of smoking prevalence it is difficult to determine the relative importance of deprivation and other characteristics in explaining inequalities in a variety of health outcomes.

Smoking prevalence in those with chronic conditions is typically lower than in the general population. This may be due to diagnosis increasing motivation to quit smoking,[35] the increase in smoking cessation advice and support [36] or the age and gender profile of those with chronic conditions. Smoking prevalence in those with chronic conditions has not reduced over the seven year period covered in this analysis, possibly suggesting that smoking cessation advice has limited effect, but this may be due to the turnover of patients with chronic conditions as a result of both premature mortality and new diagnoses. A wide range of smoking cessation advice and support has recently been reviewed by Zwar et al;[37] consideration of how these impact on those with chronic conditions is recommended as a result of this finding.

QOF smoking indicators have changed since 2004 and continue to change. The introduction, in 2012/13, of an indicator which allows estimates of the smoking prevalence within the general population is useful for researchers as well as CCGs and public health officials. The removal of the indicator that covers the recording of smoking status in the total population from QOF in 2014/15 will impact on the methodology described in this paper, although the number of

patients who are recorded as current smokers will continue to be available. The population of the practice will need to be used as the denominator in the calculation of smoking prevalence. It will be important to determine if the smoking status declines after the removal of the indicator; a recent study suggests that removal of indicators does not lead to a decline in clinical activities.[38]

Conclusion

Data published through QOF allow useful estimations of smoking prevalence within practice populations and in those with chronic conditions to be made. These estimates are important in developing our understanding of differences in health outcomes between practices, and are useful to both individual practices and CCGs when comparing practice level health outcomes, to assess need and to inform targeting. Aggregating practice level data may also be useful to allow estimates of smoking prevalence in local areas to be made. Revisions to QOF means that researchers will need to update methodology as indicators change.

For peer review only

Table 1 Summary of smoking indicators for which underlying achievement is published

General form of the indicator	Patient group	
	Patients with any, or any combination of the following conditions: coronary heart disease, stroke or TIA, hypertension, diabetes, COPD or asthma[1].	All patients aged 15 years+
**% of patients whose notes record smoking status[2]**	SM01: 2006/07 & 2007/08<br>SM03: 2008/09 – 2011/12<br>SM05: 2012/13<br>SMOK002: 2013/14 – 2014/15	Records 22: 2006/07 & 2007/08<br>Records 23: 2008/09 – 2011/12<br>SM07: 2012/13<br>SMOK001: 2013/14 – retired in 2014/15
**% of patients who are recorded as current smokers whose notes contain a record that smoking cessation advice or referral to a specialist service, where available, has been offered within the previous 15 months[3]**	SM02: 2006/07 & 2007/08<br>SM04: 2008/09 – 2011/12<br>SM06: 2012/13<br>SMOK005: 2013/14 – 2014/15	SM08: 2012/13<br>SMOK004: 2013/14 – 2014/15
**The practice supports smokers in stopping smoking by a strategy which includes providing literature and offering appropriate therapy.**		Information 5: 2006/07-2011/12<br>SMOK003: 2012/13 – 2014/15

[1]In 2008/09 CKD, asthma, schizophrenia, bipolar affective disorder or other psychoses were added to the list of chronic conditions and in 2012/13 PAD was added.
[2]For those with chronic conditions, the record must have been made in the past 15 months, reduced to 12 months in 2013/14, for all patients the period is 27 months, reduced to 24 months in 2013/14.
[3]In 2012/13 this changed to 'who have a record of an offer of support and treatment within the preceding 15 months', the period is 27 months for all patients, reduced to 12 months and 24 months respectively in 2013/14.

For peer review only

Table 2 Example of QOF data from 2012/13, showing how it can be used to calculate smoking prevalence for individual practices.

		Example practices				
QOF description	Interpretation for purposes of calculating smoking prevalence	A	B	C	D	E
SM07 Points		11	10.5	10.8	9.6	11
SM07 Numerator	Patients[1] whose notes contain a record of smoking status	3450	1319	6276	31948	6504
SM07 Denominator	Patients who are eligible to be included in this indicator[2]	3721	1497	7033	37654	7212
SM07 UA		92.70%	88.10%	89.20%	84.80%	90.20%
SM08 Points		12	9.9	12	8.9	12
SM08 Numerator	Patients who are recorded as current smokers and have a record of an offer of support etc	1024	325	1578	8439	2165
SM08 Denominator	Patients who are recorded as current smokers	1129	401	1586	10931	2373
SM08 UA		90.70%	81.00%	99.50%	77.20%	91.20%
	Calculation to determine percentage who are smokers SM08 den/ SM07 den	1129/3721	401/1497	1586/7033	10931/37654	2373/7212
	Estimate of smoking prevalence	30.30%	26.80%	22.60%	29.00%	32.90%

1[1]*Patients aged over 15*

[2]*For example patients who are newly registered with the practices (less than three months) are excluded from the indicator*

For peer review only

Table 3: Comparison of the population of each district based on the 2011 Census and aggregation QOF based practice data.

**Local authority**	Population aged 15 and over (2011 Census)[1]	Population included in QOF indicator SM07[2]	Number of general practices[3]	IHS sample size 2011/12[4]
Leicestershire				
Blaby	77600	67895	9	301
Charnwood	139800	152533	24	396
Harborough	70200	69168	8	234
Hinckley and Bosworth	87800	84159	12	305
Melton	41900	34912	2	130
North West Leicestershire	77000	78331	14	242
Oadby and Wigston	47100	48054	9	167
**Northamptonshire**				
Corby	49400	57112	5	131
Daventry	64100	71902	8	223
East Northamptonshire	70900	55279	8	217
Kettering	75900	87059	9	180
Northampton	171600	184370	27	446
South Northamptonshire	69700	60391	8	205
Wellingborough	61300	61013	9	172
**Unitary Authorities**				
Leicester	264600	293156	59	1475
Rutland	31300	29628	4	416
Totals	1400200	1434962	215	5240

[1] Data based on 2011 Census available from ONS [21]

[2] Based on QOF registers accessed from [22]

[3] Practices are matched to local authority districts based on the postcode of the practice [23].

[4] Based on IHS data 2011/12 [24]

For peer review only

Table ~~3~~4 Estimated incident rate ratios (IRRs) for premature (U75) CHD mortality count (n=215)[1].

	***without** smoking prevalence variable*			***with** smoking prevalence variable*		
**Explanatory variable**	***IRR***	***95% CI***	***p value***	***IRR***	***95% CI***	***p value***
Percentage white patients	1.007	(1.003, 1.012)	0.002	1.001	(0.995, 1.007)	0.657
Deprivation score (IMD 2007)	1.017	(1.011, 1.024)	<0.0001	1.005	(0.995, 1.015)	0.348
Prevalence of diabetes (QOF 2006/07)	1.108	(1.020, 1.203)	0.015	1.095	(1.008, 1.187)	0.031
Percentage over 65	1.060	(1.038, 1.083)	<0.0001	1.067	(1.044, 1.091)	<0.0001
Percentage male patients	1.073	(1.035, 1.111)	<0.0001	1.058	(1.021, 1.097)	0.002
Number of GPs per 1000 patients	1.209	(0.894, 1.637)	0.218	1.113	(0.821, 1.508)	0.491
Hypertension detection 2006/07 (QOF 2006/07)	0.984	(0.955, 1.014)	0.300	0.988	(0.959, 1.018)	0.416
% patients offered smoking cessation advice (SM02 - QOF 2006/07)	1.006	(0.996, 1.016)	0.271	1.010	(1.000, 1.021)	0.057
% serum cholesterol (CHD08 - QOF 2006/07)	0.989	(0.980, 0.999)	0.028	0.992	(0.983, 1.002)	0.109
% aspirin (CHD09 - QOF 2006/07)	1.007	(0.986, 1.029)	0.514	1.003	(0.982, 1.025)	0.777
% of patients with recalled perception of being able to see preferred GP (QOF 2006/07)	0.995	(0.990, 1.000)	0.069	0.995	(0.990, 1.000)	0.061
%smoking prevalence – estimated (QOF 2006/07)				1.031	(1.012, 1.052)	0.002

[1]IRR, 95% confidence intervals and associated p values as a result of negative binomial model of count of premature mortality caused by CHD.

For peer review only

Competing Interests

KH had financial support from CLAHRC in the form of funding for PhD fees. RB is in receipt of an NIHR Senior Investigator award. No financial relationships with any organisations that might have an interest in the submitted work in the previous five years; no other relationships or activities that could appear to have influenced the submitted work.

Authors' contributions

Contributor Statement

The study was conceived by KH, RB, JB and DJ. KH designed the study, carried out the analysis and drafted the initial manuscript. JB and DJ contributed to the statistical analysis. RB, JB and DJ contributed to drafting and editing the final manuscript and interpreting and reviewing the results of the ~~statistical~~ analysis.

Acknowledgements

The research was funded and led by National Institute for Health Research (NIHR) Collaboration for Leadership in Applied Health Research and Care) based at LNR. RB is in receipt of an NIHR Senior Investigator award. The views expressed are those of the authors and not necessarily those of the NHS, the NIHR or the Department of Health. The funders had no role in the study design, data collection and analysis, decision to publish or preparation of the manuscript.

Ethical committee approval

NRES advised that NHS ethics committee was not required.

Data sharing

No additional data is available

For peer review only

Bibliography

[1] Brown J. *Smoking prevalence in England below 20% for the first time in 80 years;* 2014 http://www.bmj.com/content/348/bmj.f7535/rr/683849 (accessed 3 Feb 2014)

[2] Corgan E. *Local Stop Smoking Services: Service deliver and monitoring guidance*; 2011 https://www.gov.uk/-government/uploads/system/uploads/attachment_data/file/213755/dh_125939.pdf (accessed 3 Feb 2014)

[3] Clinical Commissioning Strategy (Updated April 2013) 2012-2015; 2013. https://www.leicestercityccg.nhs.uk/-wp-content/uploads/2013/09/Commissioning-Strategy-v20.1.pdf (accessed 3 Feb 2014)

[4] Overview of CHD Programme 2013-2014;. Accessed on 8 Feb 2014. Available from: http://-www.cambridgeshireandpeterboroughccg.nhs.uk/coronary-heart-disease.htm (accessed 3 Feb 2014)

[5] Brettell R, Soljak M, Cecil E *et al*. *Reducing heart failure admission rates in England 2004-2011 are not related to changes in primary care quality: national observational study*. European Journal of Heart Failure. 2013;15(12):1335-1342 doi: 10.1093/eurjhf/hft107.

[6] Purdy S, Griffin T, Salisbury C *et al*. *Emergency admissions for coronary heart disease: a cross-sectional study of general practice, population and hospital factors in England.* Public Health. 2011;125(1):46–54.

[7] Soljak M, Calderon-Larrañaga A, Sharma P, Cecil E, Bell D, Abi-Aad G, et al. *Does higher quality primary health care reduce stroke admissions? A national cross-sectional study.* Br J Gen Pract. 2011;61(593):e801–e807.

[8] Bankart MJG, Baker R, Rashid A *et al*. *Characteristics of general practices associated with emergency admission rates to hospital: a cross-sectional study.* Emerg Med J. 2011 Jul;28(7):558–563.

[9] Kiran T, Hutchings A, Dhalla IA *et al*. *The association between cardiovascular outcomes: a cross-sectional study using data from the UK Quality and Outcomes Framework.* J Epidemiol Community Health. 2010;64:927–934.

[10] Honeyford K, Baker R, Bankart MJG *et al*. *Modelling factors in primary care quality improvement: a cross-sectional study of premature CHD mortality.* BMJ Open. 2013;3(10):e003391. doi:10.1136/bmjopen-2013-003391

[11] Twigg L, Moon G, Walker S. *The smoking epidemic in England*. London: Health Development Agency; 2004. http://www.nice.org.uk/niceMedia/documents/smoking_epidemic.pdf (accessed 10 Feb 2014).

[12] Bryant G, Chappel D, Unsworth L. *The Prevalence of Smoking in the North East - Occasional Paper No. 49*. North East Public Health Observatory; 2012.

[13] Office for National Statistics *Sample Design and Response - Appendix B - General Lifestyle Survey*. Office for National Statistics; 2011. http://www.ons.gov.uk/ons/rel/ghs/general-lifestyle-survey/2011/index.html (accessed 10 Feb 2014)

[14] Office for National Statistics. *Statistical Bulletin - Integrated Household Survey April 2010 to March 2011: Experimental Statistics.* Office for National Statistics; 2011.

[15] Blak BT, Thompson M, Dattani H, Bourke A. *Generalisability of The Health Improvement Network (THIN) database: demographics, chronic disease prevalence and mortality rates*. Inform Prim Care. 2011;19(4):251–255.

[16] QRESEARCH. The QRESEARCH database. http://www.qresearch.org/SitePages/Home.aspx (accessed 1 Feb 2014)

[17] Szatkowski L, Lewis S, McNeill A *et al*. *Can data from primary care medical records be used to monitor national smoking prevalence?* J Epidemiol Community Health. 2012;66(9):791–795.

[18] Simpson CR, Hippisley-Cox J and Sheikh A. *Trends in the epidemiology of smoking recorded in UK general practice.* British Journal of General Practice. 2010;60(572):121–127.

[19] Health and Social Care Information Centre. *Annex A: Quality indicators - Summary of points*. Health and Social Care Information Centre; 2004. http://www.hscic.gov.uk/catalogue/PUB01946 (accessed 1 Feb 2014)

[20] Health and Social Care Information Centre. *Frequently Asked Questions* http://qof.hscic.gov.uk/faqs/-index.asp#qof23 (accessed 1 Feb 2014)

[21] Office for National Statistics *Table P04 2011 Census: Usual resident population by five-year age group, local authorities in England and Wales.* 2012 http://www.ons.gov.uk/ons/publications/re-reference-tables.html?edition=tcm%3A77-257414 (accessed 3 June 2014)

[22] Health and Social Care Information Centre. *Quality and Outcomes Framework* http://www.hscic.gov.uk/qof (accessed 1 Feb 2014).

[23] Office for National Statistics *National Statistics Postcode Products* http://www.ons.gov.uk/ons/guide-method/geography/products/postcode-directories/-nspp-/index.html (accessed 3 June 2014)

[24] London Health Observatory. *Smoking prevalence among adults aged 18+ by region and local authority*. Updated August 2012 http://www.lho.org.uk/viewResource.aspx?id=16678 (accessed 3 Dec 2013)

[25] Lin LI. *A concordance correlation coefficient to evaluate reproducibility*. Biometrics. 1989;45(1):255–268.

[26] Bland JM and Altman DG. *Statistical methods for assessing agreement between two methods of clinical measurement*. Lancet. 1986;1(8476):307–310.

[27] Doran T, Kontopantelis E, Fullwood C *et al*. *Exempting dissenting patients from pay for performance schemes: retrospective analysis of exception reporting in the UK Quality and Outcomes Framework*. BMJ. 2012;344:e2405–e2405.

[28] Wong S, Shields M, Leatherdale S *et al*. *Assessment of validity of self-reported smoking status*. Health Rep. 2012;23(1):47–53.

[29] Haynes R, Lovett, A and Sünnerberg G *Potential accessibility, travel time, and consumer choice: geographical variations in general medical practice registrations in Eastern England* Environment and Panning A 2003 35(10):1733-1750

[30] Shipton D, Tappin DM, Vadiveloo T *et al*. *Reliability of self reported smoking status by pregnant women for estimating smoking prevalence: a retrospective, cross sectional study*. BMJ. 2009;339:b4347–b4347.

[31] Strong M, Maheswaran R, Pearson T. *A comparison of methods for calculating general practice level socioeconomic deprivation*. Int J Health Geogr. 2006;5(1):29.

[32] McLean G, Guthrie B, Watt G *et al*. *Practice postcode versus patient population: a comparison of data sources in England and Scotland*. Int J Health Geogr. 2008;7(1):37.

[33] Sullivan E, Baker R, Jones D *et al Primary healthcare teams' views on using mortality to review clinical policies.* Qual Saf Health Care. 2007;16:359-362

[34] Office for National Statistics: Statistical Bulletin – Integrated Household Survey April 2010 to March 2011: Experimental Statistics 2011 http://www.ons.gov.uk/ons/dcp171778_227150.pdf (accessed 3rd June 2014)

[35] Bassett JC, Gore JL, Chi, AC *et al. Impact of bladder cancer diagnosis on smoking behaviour.* J Clin Oncol 2012; 30:1871-1878

[36] Coleman T. *Do financial incentives for delivering health promotion counselling work? Analysis of smoking cessation activities stimulated by the quality and outcomes framework*. BMC public health. 2010;10(1):167.

[37] Zwar NA, Mendelsohn CP and Richmond RL *Supporting smoking cessation.* BMJ. 2014;348:f7535

[38] Kontopantelis E, Springate D, Reeves D *et al*. *Withdrawing performance indicators: retrospective analysis of general practice performance under UK Quality and Outcomes Framework*. BMJ. 2014;348:g330. Available from: http://-dx.doi.org/10.1136/bmj.g330.

For peer review only

For peer review only

Figure 1 Relationship between aggregated QOF estimates and IHS estimates for local authority districts

Legend Fig 1a Association between estimates (dashed line: estimates are equal)
Fig 1b Bland-Altman plot showing relationship between difference in estimates and mean difference (solid line: mean difference; dashed lines: 95% limits of agreement)
QOF estimates based on 2012/13 data; IHS estimates based on 2011/12 survey.

Figure 2 Relationship between QOF estimates for the general population and those with chronic conditions (2012/13)

Legend Fig 2a Association between estimates (dashed line: estimates are equal; solid line: fitted line)
Fig 2b Bland-Altman plot showing relationship between difference in estimates and mean difference (solid line: mean difference; dashed lines: 95% limits of agreement)
SM07 and SM08 (2012/13) used for QOF estimates for the general population;
SM05 and SM06 (2012/13) used for QOF estimates for those with chronic conditions.

For peer review only

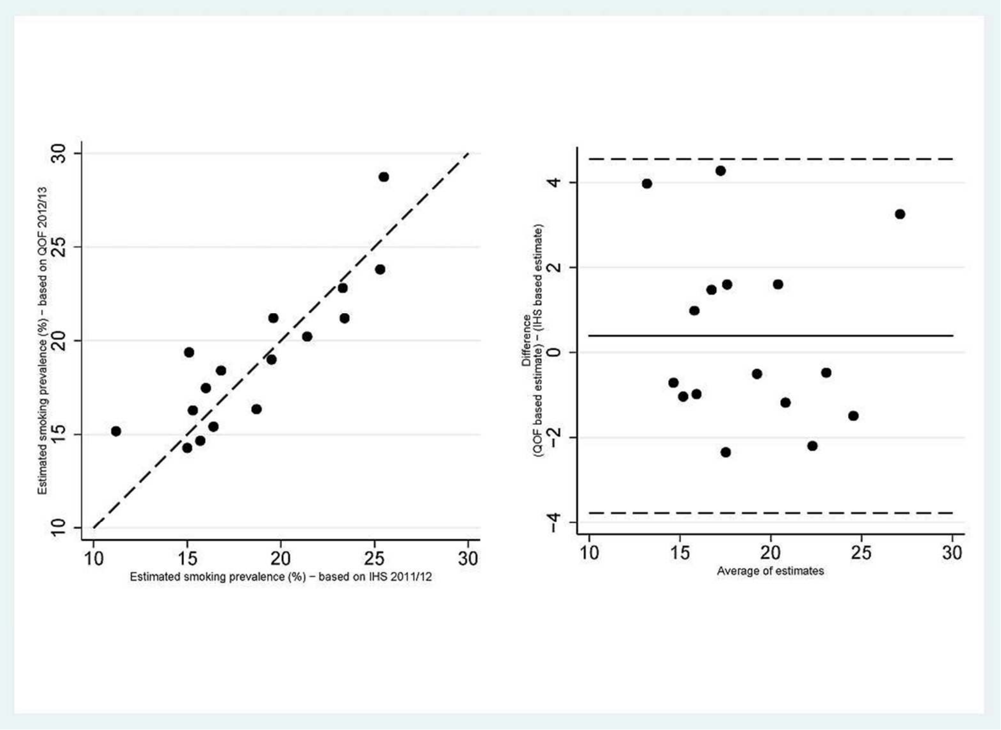

Figure 1 Relationship between aggregated QOF estimates and IHS estimates for local authority districts
Legend Fig 1a Association between estimates (dashed line: estimates are equal)
Fig 1b Bland-Altman plot showing relationship between difference in estimates and mean difference (solid line: mean difference; dashed lines: 95% limits of agreement) QOF estimates based on 2012/13 data; IHS estimates based on 2011/12 survey.
90x65mm (300 x 300 DPI)

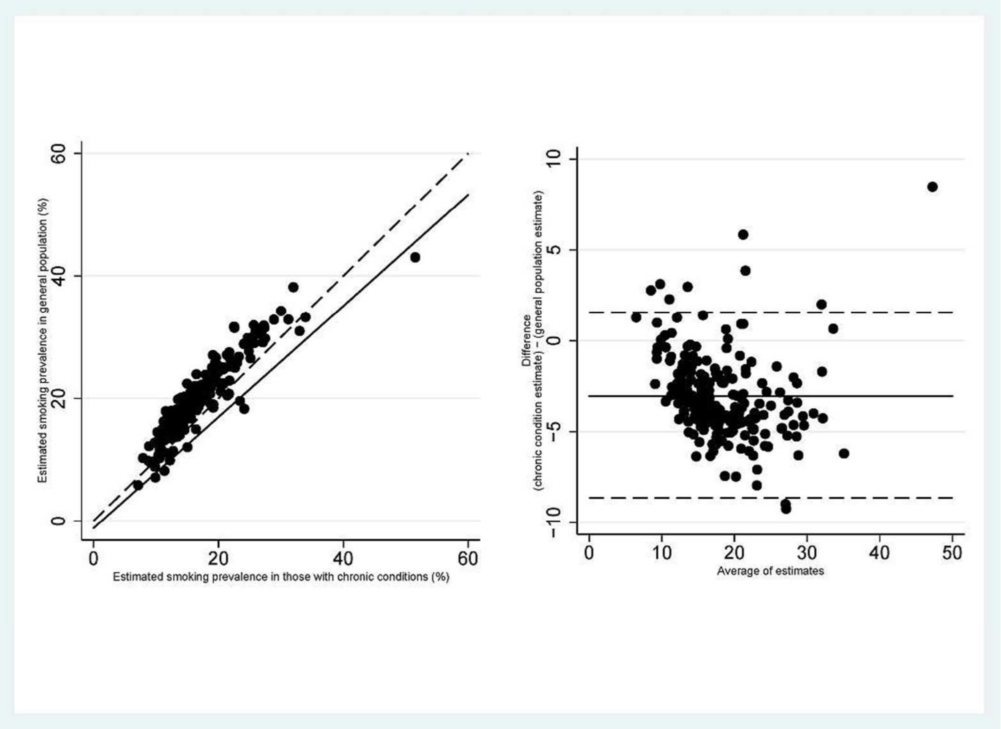

Figure 2 Relationship between QOF estimates for the general population and those with chronic conditions (2012/13)
Legend Fig 2a Association between estimates (dashed line: estimates are equal; solid line: fitted line)
Fig 2b Bland-Altman plot showing relationship between difference in estimates and mean difference (solid line: mean difference; dashed lines: 95% limits of agreement)
SM07 and SM08 (2012/13) used for QOF estimates for the general population;
SM05 and SM06 (2012/13) used for QOF estimates for those with chronic conditions.
90x65mm (300 x 300 DPI)

only

Table S1 Concordance between estimates of smoking prevalence in those with chronic conditions 2006/07 to 2012/13

Year	2012/13	2011/12	2010/11	2009/10	2008/09	2007/08
2011/12	0.97<br>*-0.15[1] (-2.4, 2.7)[2]*					
2010/11	0.97<br>*-0.12 (-2.8, 2.6)*	0.97<br>*0.04 (-2.6, 2.7)*				
2009/10	0.96<br>*-0.12 (-3.3, 3.0)*	0.96<br>*-0.03 (-3.2, 3.2)*	0.99<br>*0.00 (-2.0, 2.0)*			
2008/09	0.95<br>*0.06 (-3.5, 3.7)*	0.95)<br>*0.22 (-3.4, 3.8)*	0.97<br>*0.18 (-2.5, 2.9)*	0.98<br>*0.19 (-1.9, 2.3)*		
2007/08	0.93<br>*0.71 (-3.0, 4.6)*	0.93<br>*0.87 (-3.0, 4.8)*	0.95<br>*0.83 (-2.4, 4.0)*	0.96<br>*0.84 (-1.8, 3.5)*	0.97<br>*0.65 (-1.74, 3.0)*	
2006/07	0.93<br>*0.64 (-3.6, 4.6)*	0.92<br>*0.79 (-3.4, 5.0)*	0.94<br>*0.76 (-2.8, 4.4)*	0.94<br>*0.76 (-2.6, 4.1)*	0.95<br>*0.57 (-2.9, 4.1)*	0.97<br>*-0.08 (-2.6, 2.5)*

Lin's concordance coefficients
p<0.001 for all coefficients
Mean difference[1] and 95% Limits of Agreement[2] are given in italics

For peer review only

STROBE 2007 (v4) Statement—Checklist of items that should be included in reports of *cross-sectional studies*

Section/Topic	Item #	Recommendation	Reported on page #
**Title and abstract**	1	(*a*) Indicate the study's design with a commonly used term in the title or the abstract	3 - abstract
		(*b*) Provide in the abstract an informative and balanced summary of what was done and what was found	3 - abstract
**Introduction**			
Background/rationale	2	Explain the scientific background and rationale for the investigation being reported	4 - background
Objectives	3	State specific objectives, including any prespecified hypotheses	3 - abstract<br>4 - background
**Methods**			
Study design	4	Present key elements of study design early in the paper	3 -abstract
Setting	5	Describe the setting, locations, and relevant dates, including periods of recruitment, exposure, follow-up, and data collection	5 – methods – 'sample'
Participants	6	(*a*) Give the eligibility criteria, and the sources and methods of selection of participants	5 – methods – 'sample'
Variables	7	Clearly define all outcomes, exposures, predictors, potential confounders, and effect modifiers. Give diagnostic criteria, if applicable	5 - methods
Data sources/ measurement	8*	For each variable of interest, give sources of data and details of methods of assessment (measurement). Describe comparability of assessment methods if there is more than one group	5 - methods
Bias	9	Describe any efforts to address potential sources of bias	5 – methods & 6 – results (recording of smoking status and prevalence)
Study size	10	Explain how the study size was arrived at	5 – methods – 'sample'
Quantitative variables	11	Explain how quantitative variables were handled in the analyses. If applicable, describe which groupings were chosen and why	5 - methods

For peer review only

Statistical methods	12	(*a*) Describe all statistical methods, including those used to control for confounding	5 – methods
		(*b*) Describe any methods used to examine subgroups and interactions	5 – methods
		(*c*) Explain how missing data were addressed	5 – methods
		(*d*) If applicable, describe analytical methods taking account of sampling strategy	5 – methods
		(*e*) Describe any sensitivity analyses	6 - results
**Results**			
Participants	13*	(a) Report numbers of individuals at each stage of study—eg numbers potentially eligible, examined for eligibility, confirmed eligible, included in the study, completing follow-up, and analysed	5 - methods
		(b) Give reasons for non-participation at each stage	
		(c) Consider use of a flow diagram	
Descriptive data	14*	(a) Give characteristics of study participants (eg demographic, clinical, social) and information on exposures and potential confounders	5 - methods
		(b) Indicate number of participants with missing data for each variable of interest	
Outcome data	15*	Report numbers of outcome events or summary measures	6 - results
Main results	16	(*a*) Give unadjusted estimates and, if applicable, confounder-adjusted estimates and their precision (eg, 95% confidence interval). Make clear which confounders were adjusted for and why they were included	
		(*b*) Report category boundaries when continuous variables were categorized	n/a
		(*c*) If relevant, consider translating estimates of relative risk into absolute risk for a meaningful time period	n/a
Other analyses	17	Report other analyses done—eg analyses of subgroups and interactions, and sensitivity analyses	5 – methods<br>6 - results
**Discussion**			
Key results	18	Summarise key results with reference to study objectives	7 – discussion – principal findings
Limitations	19	Discuss limitations of the study, taking into account sources of potential bias or imprecision. Discuss both direction and magnitude of any potential bias	7 – discussion – strengths and weaknesses
Interpretation	20	Give a cautious overall interpretation of results considering objectives, limitations, multiplicity of analyses, results from similar studies, and other relevant evidence	7 - discussion
Generalisability	21	Discuss the generalisability (external validity) of the study results	7 - discussion
**Other information**			

For peer review only

Funding	22	Give the source of funding and the role of the funders for the present study and, if applicable, for the original study on which the present article is based	11 – competing interests

*Give information separately for cases and controls in case-control studies and, if applicable, for exposed and unexposed groups in cohort and cross-sectional studies.

Note: An Explanation and Elaboration article discusses each checklist item and gives methodological background and published examples of transparent reporting. The STROBE checklist is best used in conjunction with this article (freely available on the Web sites of PLoS Medicine at http://www.plosmedicine.org/, Annals of Internal Medicine at http://www.annals.org/, and Epidemiology at http://www.epidem.com/). Information on the STROBE Initiative is available at www.strobe-statement.org.

For peer review only
